# Study on the Characteristic Codon Usage Pattern in Porcine Epidemic Diarrhea Virus Genomes and Its Host Adaptation Phenotype

**DOI:** 10.3389/fmicb.2021.738082

**Published:** 2021-10-18

**Authors:** Fusheng Si, Li Jiang, Ruisong Yu, Wenqiang Wei, Zhen Li

**Affiliations:** ^1^Shanghai Key Laboratory of Agricultural Genetics and Breeding, Shanghai Engineering Research Center of Breeding Pig, Institute of Animal Science and Veterinary Medicine, Shanghai Academy of Agricultural Sciences, Shanghai, China; ^2^South China Botanical Garden, Chinese Academy of Sciences, Guangzhou, China; ^3^Department of Microbiology, School of Basic Medical Sciences, Henan University, Kaifeng, China

**Keywords:** coronavirus, PEDV, codon usage, selection pressure, host adaptation, viral evolution

## Abstract

Porcine epidemic diarrhea virus (PEDV), which classified in the genus *Alphacoronavirus*, family *Coronaviridae*, is one of the most important pathogens that cause heavy economic losses in pig industry. Although intensive mutation and recombination analysis of PEDV strains were provided, systematic genome analysis were needed to elucidate the evolution mechanism and codon usage adaptation profiles of the pathogen. Here, a comprehensive investigation was carried out to reveal the systematic evolutionary processes of synonymous codon usage and host-adapted evolution phenotype of PEDV genome. We found a low codon usage bias (CUB) in PEDV genome and that nucleotide compositions, natural selection, mutation pressure and geographical diversity shapes the codon usage patterns of PEDV, with natural selection dominated the overall codon usage bias in PEDV than the others. By using the relative codon deoptimization index (RCDI) and similarity index (SiD) analysis, we observed that genotype II PEDV strains showed the highest level of adaptation phenotype to *Sus scrofa* than another divergent clade. To the best of our knowledge, this is the first comprehensive report elaborating the codon usage and host adaptation of PEDV. The findings offer an insight into our understanding of factors involved in PEDV evolution, adaptation and fitness toward their hosts.

## Introduction

Porcine epidemic diarrhea virus (PEDV) is a pathogen causing vomiting, diarrhea, dehydration and high lethality in piglets. This pathogen was first identified in Belgium in 1978 (Pensaert and de Bouck, 1978), and since then it has been identified in other countries of the world, including China, Japan, South Korea and Thailand in Asia. Wide use of inactivated PEDV vaccine kept the incidences at minimum in China until 2010, however, emergence of new variants thereafter caused increased PEDV outbreaks with more severe morbidity and mortality in suckling piglets (Li et al., 2012), then spread to North America in 2013–2014 (Huang et al., 2013). The continuous worldwide outbreak had a huge impact on the pig industry and caused great economic losses (Jung and Saif, 2015).

PEDV belongs to the *Coronaviridae* family and is a single-stranded, positive-sense RNA virus with envelope. Its genome is about 28 kb in length and encodes 16 non-structural proteins, 4 structural proteins and an accessory protein (ORF3). Structural proteins, including spike (S), envelope (E), membrane (M), and nucleocapsid (N) proteins, are the main components of virus particles (Jung et al., 2020). The S protein is responsible for binding to cell receptor and virus invasion into host cells. The E protein is an ion channel protein involved in the virion morphogenesis (Wang et al., 2011). The M protein is the most abundant proteins in viral envelope and plays an important role in the packaging process of virus particles. The N protein combines with genomic RNA to form ribonucleoprotein (RNP), which constitutes the core of the virus particles. The only accessory protein ORF3 has ion channel activity (Wang et al., 2012), and was found beneficial to virus proliferation (Wang et al., 2012; Ye et al., 2015; Si et al., 2020). Phylogenetic analysis showed that PEDV could be divided into two genotypes (G1 and G2) and five subgenotypes (G1a, G1b, G2a, G2b, and G2c) based on complete sequence of S gene (Fan et al., 2017; Hsueh et al., 2020; Wang H. et al., 2020; Wang X.W. et al., 2020).

Synonymous codon refers to the biochemical phenomenon of codon redundancy for an amino acid. Each amino acid, in general, can be encoded by 1–6 codons. However, the patterns of codon usage in different species may be different. It was reported that the frequency of some special synonymous codons used in protein translation is significantly higher than that of other counterparts with a specific organism (Grantham et al., 1980; Martin et al., 1989; Lloyd and Sharp, 1992). The phenomenon is called as codon usage bias (CUB). CUB exists in the genomes of all species including viruses, it is regarded as a driving force of genetic evolution (Deb et al., 2021; Huang et al., 2021; Nguyen et al., 2021) and is suggested to play an important role in the adaptation of various viruses to their host (Butt et al., 2016; Kumar et al., 2018; Tian et al., 2018; Khandia et al., 2019; Luo et al., 2020a). It is also suggested that codon usage of viruses is not limited to host adaption, but is also critical in various biological processes including viral gene transcription, nuclear export of virus RNA, tolerance to translation errors and immune escape (Zhou et al., 2016; Kumar et al., 2021; Mordstein et al., 2021). In general, codon usage pattern is influenced by mutational pressure and natural selection, which continuously act on the virus coding sequences and promote the existence of codon usage preference and at last drive and optimize virus survival, fitness and continuous evolution in the host cells (Lauring et al., 2012; Luo et al., 2020b). For the reasons mentioned above, comprehensive knowledge of the related influencing factors of the codon usage patterns of viruses will benefit the study on genetic evolution and pathogenic mechanism of viruses.

While CUB is associated with a variety of biological processes and phenomena, related issues in PEDV are still open questions to be answered. Previous studies have reported the codon usage of PEDV based on its complete genome or certain genes (S and ORF3) (Cao et al., 2011; Chen et al., 2014; Xu et al., 2019; Yu et al., 2021b). However, a more comprehensive analysis is still needed to determine whether two phylogroups (G1 and G2) of PEDV differ in the codon usage patterns and other factors play an important role in shaping codon usage of PEDV. More importantly, the major bottleneck limiting our complete understanding of the ongoing PEDV outbreak is a lack of comprehensive and systematic codon usage analysis report about PEDV genome sequences, particularly those data on early epidemic strains and recent highly pathogenic mutation isolates, and the possible correlation between of them. Up to now, it is still mysterious that how PEDV codon usage pattern benefits host adaptation and viral replicative fitness, whether the codon usage pattern of the host exerts selection pressure on PEDV coding sequence or vice versa. Thus, in this study, we employed systems biology approaches to comprehensively analyze the codon usage pattern of PEDV and the corresponding influencing factors. The results of this study provided novel insight into the codon bias phenomenon and evolution mechanism of PEDV.

## Materials and Methods

### Sequence Data Retrieving and Processing

For this study, we retrieved 56 complete genomes of PEDV (recombinant sequences were excluded) from GenBank^1^ according to the isolation date, geographical distribution and phylogroups. The open reading frames (ORFs) for each genome were concatenated in the following order: ORF1ab-Spike-ORF3-Envelop-Membrane-Nucleocapsid, and each concatenated gene was subsequently retained for being analyzed on the characteristic of codon usage pattern. Detailed information about the 56 selected PEDV strains, including their isolated date, strain name, accession number and their place of isolation was listed in Supplementary Table 1.

### Phylogenetic Analysis

In order to show the genetic evolution relationship between the selected strains, phylogenetic analysis based on the non-recombinant complete nucleotide coding sequences of the 56 PEDV listed in Supplementary Table 1 was performed. The multiple sequence alignment of PEDV coding region was applied using an online tool MUSCLE.^2^ The resulting multiple sequence alignment was concatenated together to build phylogenetic trees with neighbor-joining (NJ) and maximum-likelihood (ML) algorithms, respectively. Specifically, MEGA-X software (version 10.1.8) was used to construct the phylogenetic tree with NJ method and the Kimura 2-parameter model. Nucleotide substitutions include transitions and transversions. The reliability of the phylogenetic tree was evaluated by the bootstrap methods with 1,000 replicates. IQ-TREE (version 2.1.2) (Spielman and Wilke, 2015; Minh et al., 2020) was used to perform a ML phylogenetic analysis under the TIM+F+R4 model as the best-fit model (Kalyaanamoorthy et al., 2017) using the ultrafast bootstrap option with 1,000 replicates. The phylogenetic trees were visualized using iTOL^3^ (Letunic and Bork, 2021). Bootstrap support values were labeled only if equal or larger than 50%.

### Analysis of Overall Nucleotide Composition

According to a recent study (He et al., 2021), we employed Codon W software (version 1.4.2) to compute the frequencies of A, T, C, and G at the third positions (A3s, U3s, G3s, C3s) in the synonymous codons. Meanwhile, GC contents at the first (GC1s), second (GC2s), third (GC3s) codon positions, mean of GC1s and GC2s (GC12s) and the frequencies of mononucleotides (A, C, U, and G) were calculated using R Language (version 4.0.4).^4^ The five codons, the termination codons UGA, UAG, and UAA do not encode any amino acids, AUG codons for Methionine, UGG codons for Tryptophan were excluded from the analysis.

### Relative Synonymous Codon Usage Analysis

RSCU value indicates whether the synonymous codons of a specific amino acid are used fairly or whether the codon usage pattern is affected by the amino acid composition. Usually RSCU values for the 59 codons (excluding UGA, UAG, UAA, UGG, and AUG) were computed using Codon W software (version 1.4.2) to assess the codon usage patterns. The RSCU value equal to 1 means that there is no deviation in the codon usage; while the codons with an RSCU value of < 0.6 and > 1.6 are considered as under-represented and over-represented, respectively (Sharp and Li, 1986b). If a specific codon has the highest RSCU value in both the virus and the host, this is considered as evidence of a shared codon preference (Khandia et al., 2019; Roy et al., 2021). The RSCU was calculated by the formula invented by Sharp et al. (1986):


$$RSCU_{ij}=X_{\text{ij}}/\left[\left(\frac{1}{n_{i}}\right)\sum_{j=1}^{ni}\left(X_{\text{ij}}\right)\right]$$

Where X_*ij*_ represents the number of codons used by the observed amino acid, and *n*_*i*_ represents the overall sum of synonymous codons for the amino acid.

### Correspondence Analysis of Relative Synonymous Codon Usage

Correspondence analysis (COA) is a commonly used statistical method to study the major trends of codon usage patterns in virus coding sequences, which is also known as principal component analysis (PCA) (Lara-Ramírez et al., 2014). In detail, the RSCU values of each strain were represented as a 59-dimensional vector corresponding to the 59 synonymous codons (excluding AUG and UGG encoded by single codon, as well as three stop codons UGA, UAG, UAA), and they were then transformed into uncorrelated variables (principal components) (He et al., 2019). In this method, PCA can determine the main variables according to the RSCU value of each codon and the factors influencing the CUB can be effectively determined by correspondence analysis (COA) of RSCU. The COA was conducted utilizing the Codon W software (version 1.4.2). The first two axes values which were accounting for most of the component influencing the codon usage variation among genes were used to build the PCA plots.

### Relative Dinucleotide Abundance of Porcine Epidemic Diarrhea Virus

The relative abundance of dinucleotides was calculated following a previously described method using the equation below (Kariin and Burge, 1995):


$$\rho_{XY}=\frac{f_{XY}}{f_{X}f_{Y}}$$

The odds ratio ρ_*xy*_ = *f*_*xy*_/*f*_*x*_*f*_*y*_, where the frequency of nucleotide X is represented by *f*_*x*_, the frequency of nucleotide Y is represented by *f*_*y*_, and *f*_*xy*_ denotes the observed frequency of dinucleotide *XY*. As an universal standard, for ρ_*xy*_ ≥ 1.25 or ≤ 0.78, the dinucleotide XY is over-represented or under-represented, respectively; for 1.20 ≤ ρ_*xy*_ < 1.25 or 0.78 ≤ ρ_*xy*_< 0.82, the *XY* pair is considered to be marginally high or marginally low; and for 0.82 ≤ ρ_*xy*_ ≤ 1.19, the *XY* pair is considered to be within the normal range (Kunec and Osterrieder, 2016; Khandia et al., 2019).

### Evaluation of the Influence of Selection Pressure on Codon Usage Bias

The effective codon number (ENC) analysis is used to quantify the extent of CUB of amino acid coding sequences. The ENC values ranged from 20 (only one synonymous codon for one amino acid, an extreme CUB) to 61 (all synonymous codons were used equally, showing no preference). Generally, when the ENC value is lower than 35, it is regarded as strongly biased codon usage (Comeron and Aguadé, 1998; Yu et al., 2021a). The ENC value was inversely correlated with CUB, i.e., the higher ENC value indicates the lower CUB, indicating that more synonymous codons are used to encode the same amino acid, and vice versa.

The plot of ENC vs. GC3s (the GC contents at the third position of the codon) can be utilized to determine the factors influencing the CUB (Wright, 1990). In order to determine which of the burden of mutation pressure or natural selection is the main factor affecting the codon usage of PEDV, we further analyzed the ENC-plot with the ENC values plotted against the GC3s values. If these points are located on or around Wright’s theoretical curve, the codon usage of a specific gene is only affected by mutation pressure. Otherwise, if these points are lower than the theoretical curve, natural selection plays a leading role in shaping the codon usage pattern (Wong et al., 2010). The expected ENC value was calculated by the following equation (Kumar et al., 2016):


$$ENc_{expected}=2+s+\frac{29}{s^{2}+\left(1-s^{2}\right)}$$

where s represents the frequency of G or C at the third position of synonymous codons (GC3s).

### Neutrality Plot Analysis

The neutrality plot is also called neutral evolution analysis. It is used to quantitatively measure the influences of mutation pressure and natural selection on the codon usage patterns of coding sequences (Sueoka, 1988). Using GC3s as a horizontal coordinate and GC12s (the average value of GC contents at the first and the second positions of the codons) as the vertical coordinate, the GC3s and GC12s contents of the PEDV coding sequence were plotted to create a scatterplot and a fitted regression line was calculated using R Language (version 4.0.4; see text footnote 4). The slope of regression line indicates the impact of mutational pressure (Nasrullah et al., 2015). The regression line close to the diagonal (slope = 1.0) indicates mutational pressure dominates the CUB, whereas regression curves tend toward 0 indicate natural selection plays a key role on CUB (Deb et al., 2021).

### Parity Rule 2 Analysis

Parity rule 2 (PR2) plot analysis was used to investigate the effect of mutation pressure and natural selection on the codon usage by calculating nucleotide compositions of codons at the third position (A3s, U3s, C3s, and G3s). A parity rule 2 (PR2) bias was calculated by using the AU bias [A3/(A3 + U3)] as the vertical coordinate and the GC bias [G3/(G3 + C3)] as the horizontal coordinate (Wu et al., 2015). Generally, the origin point is 0.5 (*x* = 0.5 and *y* = 0.5). If mutation pressure and natural selection have the same effect on codon usage, these points will be at the origin of the plot, where A = U and G = C.

### Hydropathicity (GRAVY) and Aromaticity Indices Analysis

GRAVY and Aroma values represent the hydropathicity and aromaticity of a given coding sequence, respectively. Those are two major factors influencing the translation and natural selection of a gene. In this study, the GRAVY and Aroma values of each strain were computed using the Codon W software (version 1.4.2) to indicate the hydropathicity and aromaticity, respectively. A higher GRAVY or ARO value suggests a more hydrophobic or aromatic amino acid product (Zang et al., 2017).

### Correlation Analysis

Correlation analysis was used to identify the relationship between nucleotide composition (A, U, C, G), the third position of code (A3s, U3s, G3s, C3s), GC3s, principal component factors, hydropathicity (GRAVY), and aromaticity (ARO) in 56 complete PEDV coding sequences using Spearman’s rank correlation analysis (Ewens and Grant, 2006).

### Relative Codon Deoptimization Index

The RCDI developed by Mueller et al. (2006) reflects the similarity between the codon usage of a gene and the codon usage of a reference genome. It can also be used to measure the rate of translation of viral gene in a host genome. Similar codon usage between a virus and its host is characterized by RCDI values close to 1, which implies that a virus is almost completely adapted to its host, and indicates that the virus follows the codon usage pattern of its host (Butt et al., 2016), whereas an RCDI higher than 1 indicates that the virus is less adaptable to the host, or the deoptimization of the codon usage patterns of the virus from that of its host (D’andrea et al., 2019). The RCDI values of different lineages of the complete PEDV coding sequence were computed using *vhcub* R package tool (Anwar et al., 2019). The codon usage patterns for *Sus scrofa* were used as references and were retrieved from the Codon Usage Database.^5^

### Similarity Index

The similarity index [SiD or D (A, B)] is employed to estimates the influence of the overall codon usage patterns of hosts on the formation of certain viruses. The range of SiD is between 0 and 1, and the higher the value, the stronger the impact of a host is on virus’s codon usage. In order to further reveal the influence of the codon usage patterns of the *Sus scrofa* on PEDV’s codon usage pattern, the similarity index was calculated as follows:


$$R\left(A,B\right)=\frac{\sum_{i=1}^{59}a_{i}\times b_{i}}{\sqrt{\sum_{i=1}^{59}a_{i}^{2}\times \sum_{i=1}^{59}b_{i}^{2}}}$$

$$D\left(A,B\right)=\frac{1-R\left(A,B\right)}{2}$$

Where *R* (A, B) is defined as the cosine value of the angle included between the A and B spatial vectors, and indicates the similarity between PEDV and the overall codon usage pattern of the host. “*a*_*i*_” is the RSCU value of a specific codon of the PEDV coding sequence, and “*b*_*i*_” is the RSCU value of the same codon for the host. *D* (A, B) indicates the potential impact of the overall use of the host codon on that of PEDV, and its value ranges from 0.0 to 1.0 (Zhou et al., 2013).

### Software and Statistical Analysis

The software Codon W (version 1.4.2) was used to calculate the overall nucleotide composition, relative synonymous codons usage (RSCU) values, correspondence analysis (COA) and the GRAVY and Aroma values of the coding sequence. Spearman’s rank correlation and linear regression analyses were performed by R Language (version 4.0.4; see text footnote 4). In some cases, the graphs were drawn by some different R packages as SeqinR and ggplot2 (Charif and Lobry, 2007; Anwar et al., 2019). An online tool CIMminer^6^ was used to performed the cluster analysis (Heat map) based on the calculated RSCU value of each PEDV strain. A *p*-value < 0.01 (^∗∗^) indicates a very significant correlation, and 0.01 < *p* < 0.05 (^∗^) indicates a significant correlation. The statistical data were analyzed by one-way ANOVA and Dunnett’s test for multiple comparisons to observe significant differences between these means from the different groups, using GraphPad Prism version 7.0 (GraphPad Software, San Diego, California, United States).

## Results

### Phylogenetic Analysis Based on Coding Sequences of Porcine Epidemic Diarrhea Virus

**FIGURE 1:**
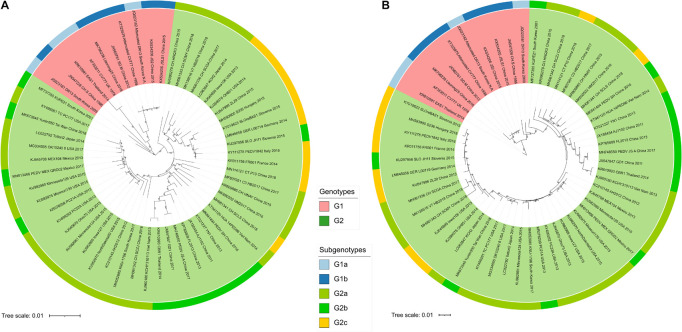
The phylogenetic trees representing the relationship of the 56 PEDV strains based on the concatenated nucleic acid alignments of 56 CDS sequences. The phylogenetic trees were generated by using the neighbor-joining **(A)** and maximum-likelihood **(B)** method, respectively. The reliability of the neighbor-joining tree was evaluated by the bootstrap methods with 1,000 replicates in MEGA-X software (version 10.1.8), whereas the maximum-likelihood phylogenetic tree was analyzed by IQ-TREE using the ultrafast bootstrap option with 1,000 replicates. Bootstrap support values were labeled only if equal or larger than 50%. The tree is drawn to scale, with branch lengths measured in the number of substitutions per site. GenBank accession numbers of strains, years, places of isolation, genogroups, and subgroups are shown.

In order to determine the relationship of PEDV strains selected in this study, we first carried out phylogenetic analysis of the complete coding sequence of PEDV by using neighbor-Joining (NJ) and maximum-likelihood (ML) algorithms method. Our results showed that the NJ and ML tree topologies were highly congruent (Figures 1A,B). It can be seen that all 56 PEDV isolates were divided into two main phylogroups (Figure 1), Group I (including G1a and G1b) and Group II (including G2a, G2b, and G2c). This was in agreement with the findings of previous reports showing two classical divergent clades (Hsueh et al., 2020; Wang X.W. et al., 2020).

### Nucleotide Composition of the Porcine Epidemic Diarrhea Virus Genome

Nucleotide content of 56 complete coding sequences was calculated to evaluate the potential impact of compositional constraints on codon usage pattern of PEDV. We found that the A%, U%, G%, C%, and GC% were 24.78 ± 0.004 (mean ± SD), 33.35 ± 0.000, 22.75 ± 0.000, 19.04 ± 0.000, and 41.79 ± 0.000, respectively. The base contents in the third position of the PEDV synonymous codons were also calculated and A3s%, U3s%, G3s%, C3s%, and GC3s% in these viruses were 23.86 ± 0.001 (mean ± SD), 54.26 ± 0.002, 22.61 ± 0.002, 22.98 ± 0.001, and 35.33 ± 0.002, respectively. We also observed that U3s (54.26%) was the highest in frequency and showed a very significant difference (*p* < 0.0001) among the A3s (23.86%), C3s (22.98%), and G3s (22.61%). In addition, the mean GC3s compositions were 35.33%, and the composition of AU (58.2%) was significantly higher than that of GC (41.79%) in PEDV complete coding sequences (*p* < 0.0001) (Supplementary Table 2), a similar trend was also observed among the five phylogroup strains (Supplementary Table 3). Taken together, these results suggest an AU-rich composition and the mononucleotide A is the most frequent nucleotide in PEDV coding sequences.

### Relative Synonymous Codon Usage Analysis

**FIGURE 2:**
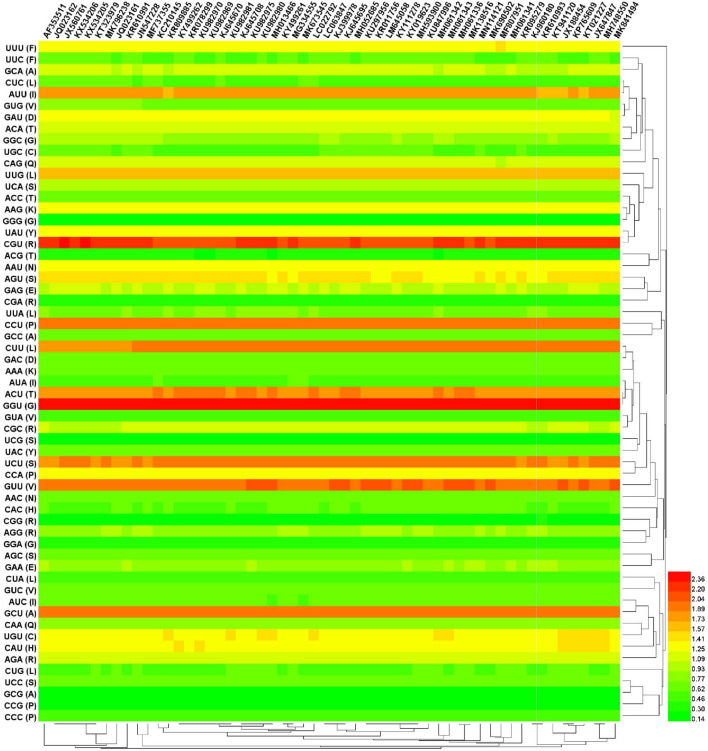
Cluster analysis (Heat map) of RSCU values among overall 56 complete coding region. The heat map represents the RSCU values divided into 3 ranges: < 1 (Green color), 1–1.6 (Yellow color), and > 1.6 (Distinct red). The heatmap analysis was performed using CIMminer. Each column represents a codon. The higher RSCU value, suggesting more frequent codon usage, was represented with distinct red. The codon usage is highly biased toward A/U-ending codons. Euclidean distance and complete-linkage methods were used to produce the clusters.

RSCU value is not related to amino acid composition, and has been widely used to evaluate the bias of codon use among genes. The higher the RSCU value means the higher the frequency of codon use or the higher the CUB phenomenon. In general, the RSCU values of the majority of the codons scored between 0.6 and 1.6. To investigate the codon usage patterns and the extent of CUB in the PEDV complete coding sequences, the RSCU values were calculated for each strain and compared with its natural host and other reference animal model hosts including Homo sapiens (Table 1). We observed that all the analyzed 18 amino acids had multiple synonymous codons (Table 1 and Figure 2). Specifically, among 59 synonymous codons, 26 were classified as preferred codons (RSCU > 1.0), and 21 of them are A/U-ended. Among preferred codon, 10 codons GCU (Ala), UUG (Leu), ACU (Thr), CCU (Pro), UCU (Ser), AUU (Ile), CUU (Leu), GUU (Val), CGU (Arg), and GGU (Gly) with RSCU value of > 1.6 were considered over-represented. Similarly, 11 codons GCG (Ala), CUA (Leu), GUA (Val), CGA (Arg), CCG (Pro), GGA (Gly), UCG (Ser), CCC (Pro), ACG (Thr), CGG (Arg), and GGG (Gly) with RSCU value of < 0.6 were regarded as under-represented and 7 out of 11 under-represented codons were G/C-ended. It is quite interesting to note that almost all of the over-represented codons were U-ended (9 out of 10) and mostly under-represented codons were A/G-ended (10 out of 11) (Table 1). We could not find a common single codon, which was over-represented in PEDV and the three model species. However, we observed several coincident preferred codons of PEDV and *Sus scrofa*; In contrast, 6 codons, CUA (Leu), GUA (Val), UCG (Ser), CCG (Pro), ACG (Thr), GCG (Ala), were under-represented not only in PEDV but also in other three model species. In addition, there were 2 codons, GUU (Val) and CGU (Arg), which were over-represented in PEDV but not the reference animal hosts species (Table 1). This result implied that PEDV had evolved a mixture of coincident and antagonistic codon usage patterns relative to its natural host, *Sus scrofa*. When clustering these biases according to a heat map, we also observed that all the PEDV strains from distinct phylogroups or different geographical areas shared similar preferred codons as above (Table 2 and Supplementary Figures 1A,B). The result indicated that CUB existed in PEDV genomes and A/U-ended codons were preferred.

**TABLE 1:**
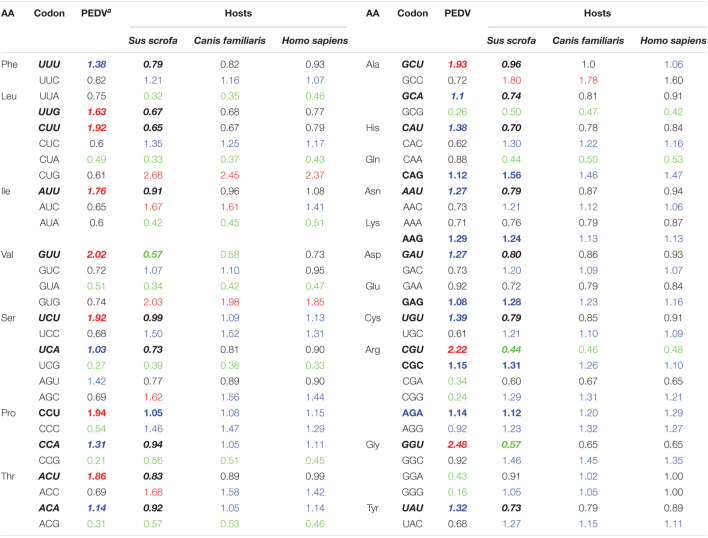
The relative synonymous codon usage (RSCU) patterns of PEDV in comparison with the RSCU values of its natural host (*Sus scrofa*) as well as reference animal model hosts including Homo sapiens.

*^a^Mean RSCU values of 56 PEDV strains. Termination codons (TER) and single codons encoding methionine (Met) and Tryptophan (Trp) were excluded from correspondence analysis. Blue, red, and green RSCU values represent the preferred (RSCU > 1), over-represented (RSCU > 1.6), and under-represented (RSCU < 0.6) codons, respectively. Coincident preferred codon of PEDV and Sus scrofa are shown in bold, most preferred codons in PEDV displaying antagonism with Sus scrofa are marked in bold italic.*

**TABLE 2:**
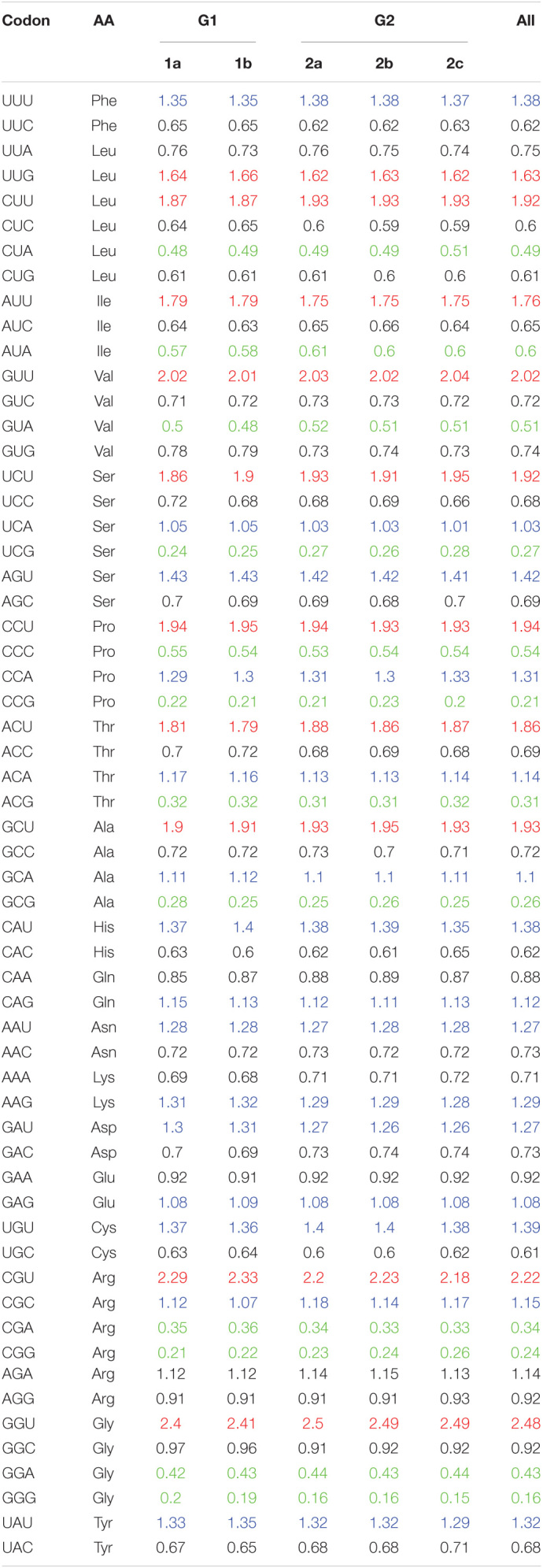
RSCU values of the whole coding sequence in 56 PEDV strains.

*Note: The values are presented according to genotypes. Blue, red, and green represent the preferred (RSCU > 1), over-represented (RSCU > 1.6), and under-represented (RSCU < 0.6) codons, respectively.*

### Correspondence Analysis

**FIGURE 3:**
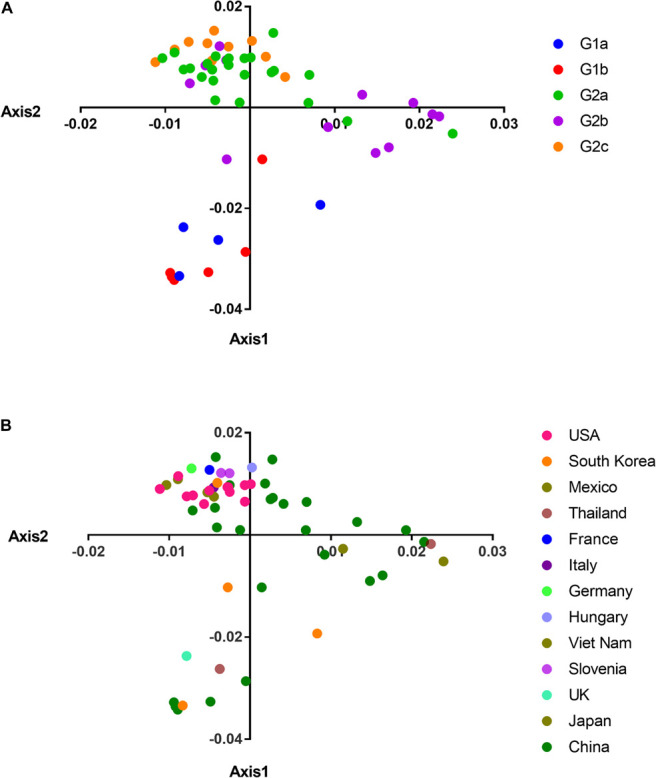
COA on the RSCU values. **(A)** PCA plot according to separate clades. **(B)** PCA plot data according to country. The clades and countries are represented in different color. The analysis was based on the RSCU value of the 59 synonymous codons. The positions of each virus were described in the first two main-dimensional coordinates.

In order to explore whether the phylogroup and geographic distribution had an impact on the codon usage pattern, we used COA based on the RSCU values to analyze the codon usage of different PEDV isolates which were isolated from different geographic areas. The result showed that the first, second, third, and fourth axis accounted for 34.81, 12.98, 10.01, and 7.97% of the total variation, respectively. This indicated that the first and second axes were responsible for the main change in the variation of the CUB which was further plotted to understand the distribution of synonymous codons usage patterns. The COA results showed that axis 2 separated G1 from the G2 genotype of PEDV (Figure 3A), which is in consistent with the phylogenetic analysis. When taking the geographical factors that may potentially influence PEDV evolution into consideration, we found that there was an obvious geographical distribution. As shown in Figure 3B, most of the isolates from different geographical areas were distributed separately except the strains from the United States, which concentrated on the top of the ordinate axis. In addition, we can see clearly that the PEDV strains from China were diversely distributed in three independent areas among the strains, which demonstrated that different strains from the same geographical region had great differences in their codon usage. These results indicate that the geographical diversity may influence PEDV CUB potentially.

### Relationship Between Relative Dinucleotide Abundance and Codon Usage in Porcine Epidemic Diarrhea Virus Genome

**FIGURE 4:**
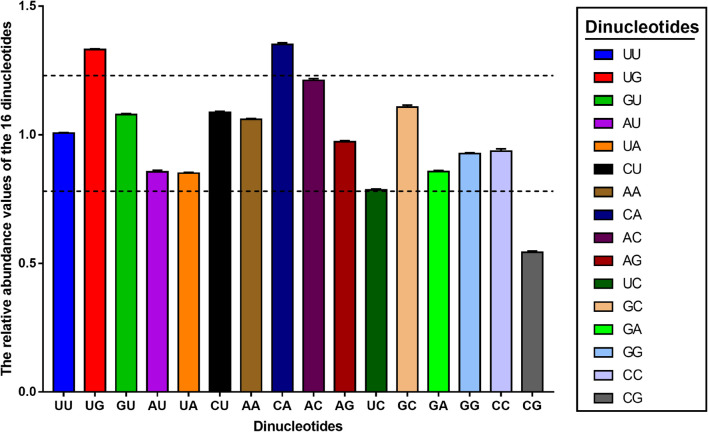
The relative dinucleotide abundance values of the PEDV complete coding sequence. The different colors represent the different dinucleotides, the above and below dashed lines represent 1.25 and 0.78, respectively. Dinucleotides are regarded as under-represented or over-represented if the relative abundance values are below 0.78 or over 1.25 (dashed lines), respectively.

It was reported that the codon usage pattern in several organisms, including some of the DNA and RNA viruses could be constrained by the relative abundance of dinucleotides (Kariin and Burge, 1995; Nasrullah et al., 2015; Wang et al., 2016; Cheng et al., 2020), which may be a consequence of intrinsic characteristics of the virus or mutational pressure from the host. Thus, we were interested in determining the relative abundance of 16 dinucleotides for all PEDV sequences. Results showed that distribution of relative dinucleotides abundance was not random in PEDV coding region (Figure 4 and Supplementary Table 4). Specifically, dinucleotides UU, GU, CU, and AA were marginally consistent with the theoretical value (1.006 ± 0.002, 1.078 ± 0.004, 1.078 ± 0.004, and 1.059 ± 0.004, respectively), dinucleotides UG and CA were over-represented (ρ_*xy*_ ≥ 1.25, ρ_*xy*_ = 1.331 ± 0.003, and ρ_*xy*_ = 1.351 ± 0.006), dinucleotide AC was close to over-represented (ρ_*xy*_ = 1.211 ± 0.007), dinucleotide CG was under-represented (ρ_*xy*_ ≤ 0.77, ρ_*xy*_ = 0.543 ± 0.005). These observations suggested that PEDV genome had a unique dinucleotide usage pattern. Taken together, our results suggested that dinucleotide composition also played a role in shaping the synonymous codon usage pattern of PEDV.

### Overall Extent of Codon Usage Bias in Porcine Epidemic Diarrhea Virus

**FIGURE 5:**
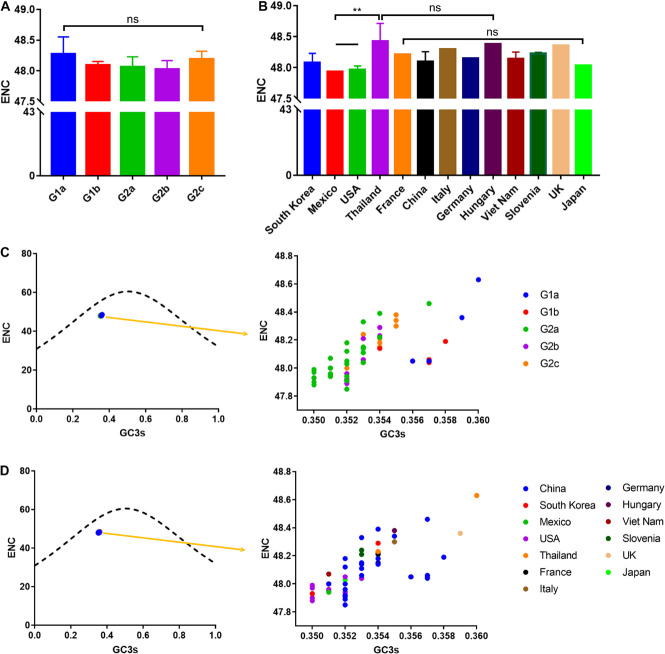
ENC and ENC-plot analysis (GC3s plotted against ENC) of the PEDV complete coding sequence. **(A)** ENC value classified according to separate clades. **(B)** ENC value classified according to isolation nation. The relationships between effective number of codons (ENC) values and GC contents at the third codon position (GC3s) of synonymous codons are represented. **(C)** Points classified by phylogroups. **(D)** Points classified by isolation nation. The ENC of all PEDV isolates was clustered slightly below the solid curved line indicating selection pressure play a role on the PEDV codon usage pattern. Asterisk indicated the differential ENC value of PEDV between the indicated groups is significant (*p* < 0.05). ns, not significant, *p* > 0.05.

RNA viruses usually have a high ENC value profile, which helps the virus replication and facilitates the adaptation of the host to the preferred codons. The average ENC value of RNA viruses are estimated to be between 38.9 and 58.3 (Jenkins and Holmes, 2003). In this study, we observed that the ENC values for all the studied PEDV isolates ranged from 47.85 to 48.63 (Supplementary Table 2). In addition, the ENC value showed no statistically significant difference among different phylogroups, with 48.27 ± 0.270 in G1a, 48.09 ± 0.061 in G1b, 48.07 ± 0.162 in G2a, 48.03 ± 0.139 in G2b, 48.19 ± 0.132 in G2c, and 48.1 ± 0.166 in all of the 56 calculated isolates (*p* > 0.05) (Figure 5A). Furthermore, when considering the ENC values by different nations, we observed that the epidemic strains in Thailand had a significantly high value than the US and Mexico strains (Figure 5B). Those results proved that a low CUB existed in the PEDV genome.

In order to further study the influence of mutational pressure on the CUB pattern of PEDV genome, we then evaluated the correlation between the nucleotide compositions (A%, U%, G%, C%, and GC%), codon compositions (A3s, U3s, G3s, C3s, and GC3s) and ENC values (Table 3). The results indicated that most of the codon compositions correlated or significantly correlated with the nucleotide compositions. A3s content has a significant negative correlation with the contents of G and GC, but a positive correlation with that of U, GC2s and GC12s. U3s content has a significant negative correlation with the content of G, C, GC, and ENC, but a positive correlation with that of U. G3s content has a significant negative correlation with the contents of U, GC2s, and GC12s, but a positive correlation with that of G, GC, and ENC. C3s content has a significant negative correlation with the content of U, but a positive correlation with that of G, C, GC, and ENC. GC3s content has a significant negative correlation with the content of U, but a positive correlation with that of G, C, GC, and ENC. These results proved that the CUB of the PEDV was influenced by nucleotide compositions, which further confirmed that the mutational pressure has contributed in shaping the codon usage patterns within PEDV genomes.

**TABLE 3:** Correlation analysis between the codon compositions (A3s, U3s, G3s, C3s, and GC3s), the ENC values, nucleotide compositions (A%, U%, G%, C%, and GC%) of the 56 PEDV strains.

	A3s	U3s	G3s	C3s	GC3s
A	0.109	–0.069	0.038	–0.017	0.007
U	0.278*	0.906**	−0.471**	−0.880**	−0.806**
G	−0.929**	−0.698**	0.948**	0.447**	0.872**
C	0.192	−0.586**	–0.145	0.773**	0.348**
GC	−0.503**	−0.865**	0.610**	0.792**	0.844**
GC1s	0.257	0.108	–0.212	–0.087	–0.158
GC2s	0.306*	–0.111	−0.411**	0.258	–0.105
GC12s	0.376**	0.028	−0.396**	0.068	–0.187
ENC	0.03	−0.858**	0.268*	0.859**	0.666**

*The numbers in each column represent correlation coefficient “r” values, which are calculated in each correlation analysis.*

“*” means 0.01 < P < 0.05; “**” means P < 0.01.

### Effective Codon Number Plot Analysis

Given that the relatively low CUB in the PEDV genome described above, this promotes us to clarify which factors affecting PEDV CUB. We then assessed the relationship between the ENC value and the percentage of G or C in the third site of codons (GC3s) in PEDV genomes. In ENC vs. GC3s graph, the curve represents the theoretically expected ENC values only considering the mutation factors and the points represents the actual ENC values which were calculated in the current study (Figures 5C,D). According to the ENC-GC3s plots, all obtained points of the PEDV coding sequence are below the expected ENC curve, which indicates that the effective codon usage for all of 56 complete coding sequences is lower than expected. Therefore, it can be explained that, in addition to mutation pressure, natural selection also plays a role in shaping the codon usage pattern of the PEDV genome.

### Neutrality Plot Analysis

**FIGURE 6:**
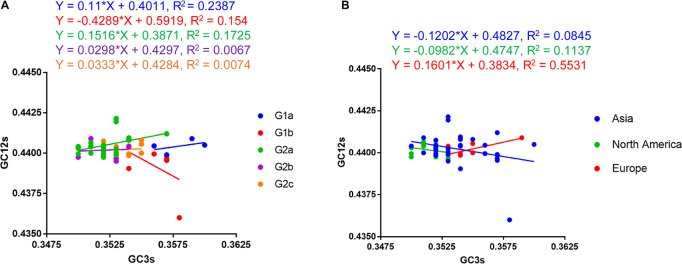
Neutrality plot analysis (GC12s against GC3s) of the PEDV complete coding genes. **(A)** Plot according to phylogroups. **(B)** Plot according to continent. The regression line is represented by the corresponding straight line, and the regression equation is also shown. The slope value indicates the mutational pressure. The neutrality plot shows the correlation between GC content in synonymous positions (GC12s) and GC content in non-synonymous positions (GC3s).

A neutrality plot analysis is a method to quantify mutational pressure and natural selection, which are two main evolutionary forces on a gene or genome. In order to know which factor plays a dominant role in shaping the codon usage pattern of PEDV coding sequence, we performed a neutrality plot analysis. We plotted GC12s as the vertical coordinate and GC3s as the horizontal coordinate to draw a linear regression line (Figure 6). In general, if the nucleotide changes at the third position of the codon don’t influence the deduced amino acids, they are just viewed as a mutational pressure. In the meantime, nucleotide changes that cause amino acid changes are considered a selection pressure. As shown in Figure 6A, a positive correlation was observed between the GC12s and GC3s values for G1a and G2 PEDV strains except G1b strains. The slopes of the linear regression were 0.11, −0.4289, 0.1516, 0.0298, and 0.0333 for G1a, G1b, G2a, G2b, and G2c coding sequences, respectively. These results indicate that mutational pressure accounted for 11, 15.16, 2.98, and 3.33% of the selection force for the G1a, G2a, G2b, and G2c coding sequences, whereas natural selection accounted for 89, 84.84, 97.02, and 96.67%, respectively. When considering the strains from different geographical areas, we also observed that natural selection accounted for 87.98% in Asia strains, 90.18% in North America strains and 83.99% in Europe strains, respectively (Figure 6B). Hence, neutrality analysis indicated that natural selection is the main force in shaping the CUB of PEDV.

### Parity Rule 2 Bias Plot Analysis

**FIGURE 7:**
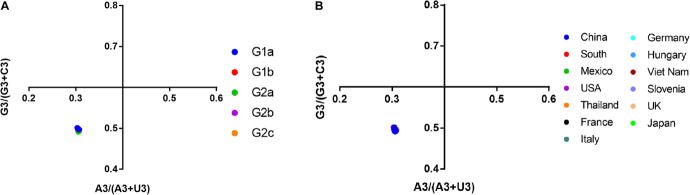
Parity Rule 2 (PR2)-bias plot [A3/(A3 + U3) against G3/(G3 + C3)]. The PR2 bias plot was calculated for the PEDV complete coding sequence. G3/(G3 + C3) and A3/(A3 + U3) are horizontal and vertical axes, respectively. **(A)** Plot classified by phylogroups. **(B)** Plot classified by country.

A PR2 bias plot analysis is another tool to study the effect of mutational pressure and natural selection on the CUB of genes. Proportional distribution of bases indicates function of mutational pressure in affecting CUB in the virus genome. On the contrary, the disproportionate distribution of bases suggests the role of both mutational pressure and natural selection in determining the CUB (Sueoka, 1995). We analyzed the relationship between the purine (A and G) and the pyrimidine (C and U) content, with [A3/(A3 + U3)] on the vertical coordinate and [G3/(G3 + C3)] on the horizontal coordinate (Figure 7). Our results showed the means of AU bias [A3/(A3 + U3)] and GC bias [G3/(G3 + C3)], which were 0.3054 (0.3054 ± 0.001) and 0.4959 (0.4959 ± 0.002), respectively (Supplementary Table 5). A bias value larger than 0.5 suggests a preference for pyrimidine over purine (Zhang et al., 2018). Thus in PEDV, U is preferred over A, while C is preferred over G in the third codon position, regardless of PEDV strains from different phylogroups (Figure 7A) or different geographic locations (Figure 7B). This result suggested that both mutational bias and natural selection might have shaped the CUB in PEDV genomes.

### Correlation Analysis Results

We also conducted the correlation analysis between the codon compositions, the first two principal axes value and the general average hydropathicity (GRAVY) and aromaticity (ARO) values. Our results revealed that these compositions were significantly correlated with the first axis, but not the second axis (Table 4). We also proved that ENC values had a negative correlation with the hydrophobicity (*r* = −0.353, *p* < 0.01), whereas there is no significant correlation between the ENC value and the aromaticity (*r* = 0.023, *p* > 0.05) (Table 5). In addition, the correlation analysis based on the PEDV complete coding sequences indicated that GRAVY is negatively correlated and significantly negatively associated with C3 (*r* = −0.331, *p* < 0.05) and ENC (*r* = −0.353, *p* < 0.01), respectively. Whereas, ARO showed a positive correlation with G3 (*r* = 0.339, *p* < 0.05) but was negatively correlated with A3 (*r* = −0.373, *p* < 0.01) and Axis1 (*r* = −0.445, *p* < 0.01) (Table 5). These results indicate that the overall average aromaticity and hydrophilicity of PEDV genome are related to the CUB, indicating that natural selection pressure has an effect on the codon usage pattern of PEDV. All together, these observations proposed that other factors, such as natural selection and nucleotide compositions, might also involve in shaping the CUB of PEDV.

**TABLE 4:** Correlation analysis between the nucleotide compositions and the two principal component axes in PEDV genomes.

Base composition	Axis1	Axis2
A	–0.001	–0.173
U	0.389**	0.061
G	−0.892**	–0.033
C	0.199	–0.010
GC	−0.541**	0.007
A3s	0.828**	0.005
U3s	0.481**	0.184
G3s	−0.891**	–0.123
C3s	–0.228	–0.203
GC3s	−0.700**	–0.206
ENC	–0.117	–0.046

*The numbers in each column represent correlation coefficient “r” values, which are calculated in each correlation analysis.*

“**” means P < 0.01.

**TABLE 5:** Correlation analysis among ARO, GRAVY, the first two axes, GC3s, ENC and GC in the 56 PEDV strains.

		Axis1	Axis2	ENC	GC	A3s	U3s	G3s	C3s	GC3s
GRAVY	*r*	–0.198	–0.158	−0.353**	–0.203	–0.180	0.137	0.242	−0.331*	–0.015
	*p*	0.143	0.243	0.008	0.133	0.185	0.313	0.073	0.013	0.915
ARO	*r*	−0.445**	0.273	0.023	0.161	−0.373**	–0.123	0.339*	0.118	0.237
	*p*	0.001	0.042*	0.866	0.236	0.005	0.367	0.011	0.386	0.079

The numbers in each column represent correlation coefficient “r” values, which are calculated in each correlation analysis. “*” means 0.01 < P < 0.05; “**” means P < 0.01.

### Genotype I Porcine Epidemic Diarrhea virus strains Showed the Highest Host Adaptation Phenotype for *Sus scrofa*

**FIGURE 8:**
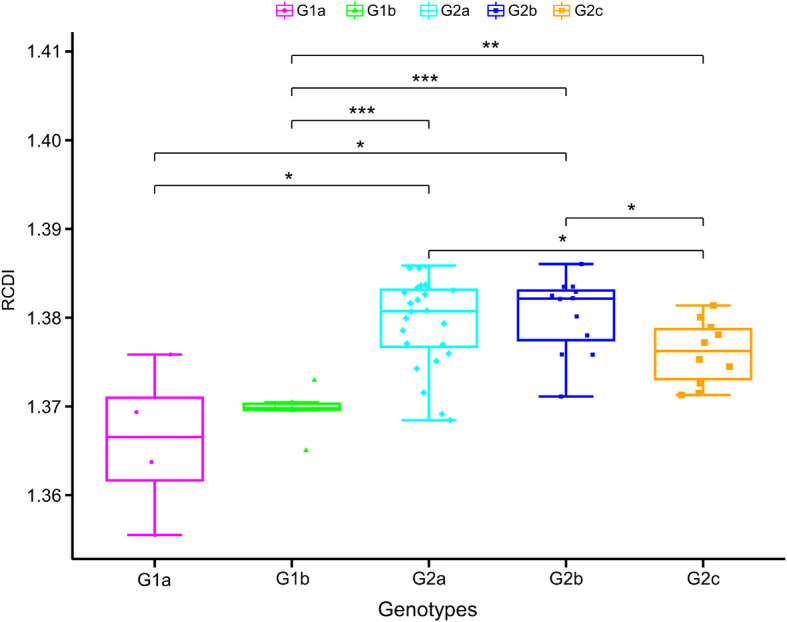
Measures of PEDV adaptation in *Sus scrofa*. The relative codon deoptimization index (RCDI) analysis of PEDV coding sequences in relation to its hosts. In the plot, the abscissa represents the phylogroups, the ordinate represents the RCDI value. One-way ANOVA and Dunnett’s test were employed to compare the mean of the RCDI values pertaining to the different phylogroups. Asterisk indicated the differential RCDI value of PEDV between G1 and G2 is statistically significant or very significant (*p* < 0.001 or *p* < 0.0001).

Relative codon deoptimization index (RCDI) values are measured by comparing the codon usage of virus with that of its host. A lower RCDI value indicates higher adaptation of a virus to its host. Conversely, a high RCDI value indicates that the virus is less adaptive to its host. In the cases of PEDV, the mean RCDI for genotype I strains was found statistically significantly lower compared with genotype II strains (Figure 8), which suggest that genotype I PEDV strains have a higher degree of adaptation to *Sus scrofa*.

### *Sus scrofa* Exerted a Significantly Deeper Selection Pressure on Porcine Epidemic Diarrhea Virus Genotype II Strains

**FIGURE 9:**
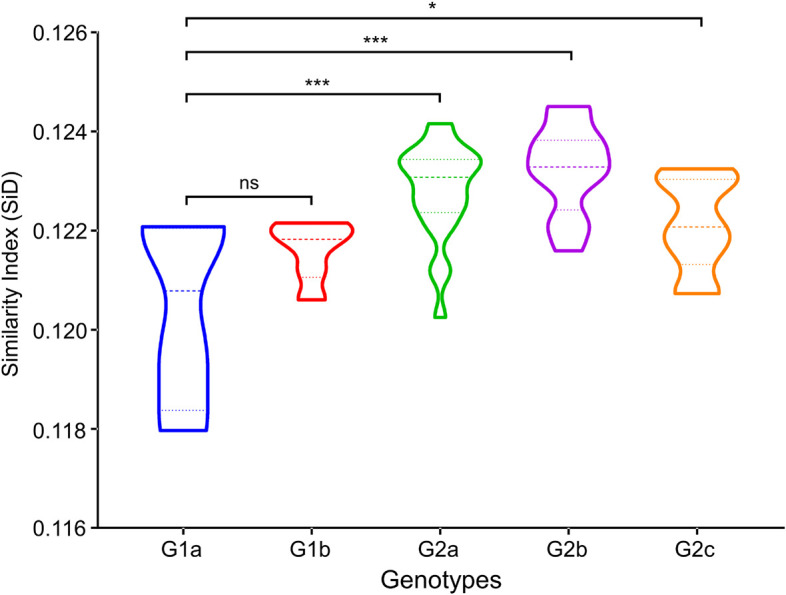
Similarity index (SiD) analysis of complete genomic coding sequences of PEDV genome in relation to the host (*Sus scrofa*). In the plot, the abscissa represents the phylogroups, the ordinate represents the SiD value. One-way ANOVA and Dunnett’s test were employed to compare the mean of the D (A, B) values pertaining to *Sus scrofa*. Asterisk indicated the differential RCDI value of PEDV between G1 and G2 is statistically significant or very significant (*p* < 0.001 or *p* < 0.0001), ns, not significant, *p* > 0.05.

SiD analysis allowed for a direct measurement of the codon usage similarities between the hosts and viruses. SiD analysis was carried out to demonstrate whether the host (*Sus scrofa*) influences the codon usage patterns of the PEDV in the process of evolution and which phylogroup was most affected. It was observed that the mean SiD value was highest in genotype II strains regardless of grouped by phylogroups (Figure 9). Specifically, SiD value of subgenotype 1a (0.1204 ± 0.0002) was statistically significantly lower compared with subgenotypes 2a (0.1228 ± 0.0010), subgenotypes 2b (0.1232 ± 0.0010), and subgenotypes 2c (0.1221 ± 0.0010) (Figure 9). This relatively low D (A, B) values indicated that Genotype I PEDV strains can replicate more efficiently in the *Sus scrofa* system without much impact on the host codon usage. Taken together, these results suggested that *Sus scrofa* induced a significantly stronger selection pressure on Genotype II strains, which implied that genotype I strains might be more adapted to their host (*Sus scrofa*) than genotype II strains.

## Discussion

Codon usage bias (CUB) refers to the unbalanced use of codons when encoding an amino acid. Mutation pressure and natural selection are two main factors affecting CUB in a species genome (Sharp and Li, 1986a; Sharp et al., 1986; Karlin and Mrázek, 1996; Barbhuiya et al., 2020), but other factors such as base composition, evolutionary pressure and geographic distribution may also have influence. While extensive studies on nucleotide composition property and CUB of advanced life forms such as *Drosophila* and mammals have been carried out (Eyre-Walker, 1991; Machado et al., 2020), similar studies with pathogens are limited. It is believed that knowledge about the codon usage patterns and the related influencing factors is important to understand the genetic evolution of the pathogens, such as bacteria and viruses (Cai et al., 2009; van Hemert et al., 2016; Zhou et al., 2019; Jin et al., 2020; MacLean et al., 2021). To fill in the gaps in the study of PEDV, we adopted several analytical approaches in this study to investigate the codon usage pattern and elucidate the involved factors influencing PEDV codon bias.

Relative synonymous codon usage (RSCU) analysis is the most commonly used tool to study the CUB of a gene. The codon usage patterns are specific to family, genus and even at the species level. In order to analyze this specificity in detail at the species level, the RSCU values of PEDV were computed and compared with host species. According to the values observed in this study, PEDV showed a CUB phenomenon in its genome, since out of the 26 preferred codons (except UGG, AUG, and stop codons) 5 were A-ended, 16 was U-ended, 4 were G-ended, 1 was C-ended. The content of A/U is the highest in PEDV genome (mean value was 24.78 and 33.35, respectively). This result indicated that PEDV prefer A/U-ended codons, further proving existence of CUB in PEDV genome. Among the RSCU values observed, almost all of the over-represented codons are U-ended, and the majority under-represented codons are A/G-ended. It is worth to note that the 6 codons, CUA (Leu), GUA (Val), UCG (Ser), CCG (Pro), ACG (Thr), GCG (Ala), were under-represented in PEDV and all the reference hosts species. Furthermore, almost all the RSCU values of less than 0.5 in those under-represented codons were presented as NCG/CGN form, indicating a strong CpG suppression or deficiency (Table 1). Study has shown that during the process of evolution, many viruses tended to reduce the content of CpG in their genomic components, which benefits its immune escape or host adaption. For example, the extremely low CpG dinucleotide content favorited influenza B virus to adapt to its human host (Greenbaum et al., 2008). The avoidance of the CpG dinucleotide is also commonly observed in many other RNA viruses (Kunec and Osterrieder, 2016; Wang et al., 2016; Roy et al., 2021), and is regarded as another selective pressure contributing in CUB (Gómez et al., 2011; Kumar et al., 2016). According to reports, the unmethylated CpGs of viral pathogens can be recognized by Toll like receptor 9 (TLR9) in the host cell, thereby generating an immune response to combat the pathogen (Dorn and Kippenberger, 2008; Kunec and Osterrieder, 2016). Thus, CpG deficiency in PEDV coding sequence appears to be an effective strategy to escape the host antiviral immune response, which reminds us that we should take this phenomenon seriously during the formulation and implementation of anti-PEDV strategies. Indeed, recently studies proved that this strategy was employed in other human and swine derived coronavirus, i.e., the novel severe acute respiratory syndrome coronavirus 2 (SARS-CoV-2) and transmissible gastroenteritis virus (TGEV) (Cheng et al., 2020; Roy et al., 2021). It is suggested that CpG deficiency in RNA viral genomes is another selective pressure contributing in CUB (Vetsigian and Goldenfeld, 2009; Gómez et al., 2011). In our study, this CpG deficiency composition in PEDV and perhaps other RNA viruses, has the potential possibility to benefit the viruses to adapt to their host and escape the host’s defense system. Studies have shown that RNA viruses generally have low CUB, and their ENC values are in the range of 47.62–57.23 (Hu et al., 2011; Lara-Ramírez et al., 2014; Cristina et al., 2015; Zhou et al., 2019; Nguyen et al., 2021), which would facilitate virus replication and adaptation to the host immune system (Chen et al., 2014; Khandia et al., 2019). In order to prove whether the CUB in PEDV genome follows this rule, we calculated the ENC value in each genome and the results in this study indicated that there is a low CUB in all PEDV isolates with the mean ENC value of 48.1 (Supplementary Table 2), this value falls in the low bias range and was compatible with the similar study on PEDV (Chen et al., 2014). We also compared the difference of ENC values between G1 and G2 phylogroups and found that ENC value has little change between the two phylogroups. The low CUB may be important for the efficient replication of PEDV in the host cells, and support the consensus that RNA virus genome sequences are prone to mutation in the process of evolution.

It is believed that the codon usage pattern is mainly affected by mutation pressure and natural selection, but it is still unknown what the case for PEDV. According to Wright’s method (Wright, 1990), we employed an ENC vs. GC3s analysis to evaluate the selection pressure on the PEDV codon usage pattern. Our results demonstrated that the data points representing the ENC value for each PEDV isolate were located below the expected curve and clustered together, suggesting that natural selection plays a role in PEDV codon usage pattern. Besides, neutrality plot analysis was conducted to quantify the effect of mutational pressure and natural selection and results supported that natural selection plays an important role in shaping the codon usage of PEDV. Moreover, Parity rule 2 (PR2) analysis showed that U and C were preferred over A and G, implying that PEDV CUB was shaped by mutation pressure and natural selection.

We also found a significant strong correlation between the overall composition of majority nucleotides and the composition of the third position in the codon (Table 3), which confirmed the effect of mutational pressure on CUB according to a previous study (Chen et al., 2014). In addition, the correlation analysis also showed that there was a significant correlation of the ENC values with the overall nucleotide content and axis value from principal component analysis (PCA), and the GRAVY/ARO values with the third nucleotide position of codon, which further supported the influencing role of mutational pressure and the effect of natural selection on PEDV CUB, respectively. It is worth to note that COA analysis found that two phylogroups of PEDV clustered separately between the first two axes, indicating that the codon usage pattern between different phylogroups have difference. Apart from those two selection pressures, reports have showed that other factors such as geographic distribution (Chen et al., 2014; Nguyen et al., 2021) and relative dinucleotide frequencies (Cheng et al., 2020; Munjal et al., 2020) also acts as the driving forces in shaping the codon usage pattern in many RNA viruses. Specifically in our study, unique dinucleotide usage pattern and the relative dinucleotide frequencies show that dinucleotide composition also plays a role in the synonymous codon usage pattern of PEDV. Moreover, COA analysis revealed that the geographical diversity may influence the entire PEDV codon usage, which reflects the evolutionary characteristics of PEDV genome to some extent. In this case, the CUB may be one of the potential factors driving the evolution of PEDV.

The RCDI has been suggested to be an effective index of the degree of viral adaptation to a host’s cellular environment. Among the two classical divergent clades, genotype I strains presented statistically significantly lower RCDI value compared with genotype II strains (Figure 8), suggesting more adapted potential and fitness of PEDV genotype I strains to swine cellular system. Because there is no reference in this regard, we are concerned that a single RCDI value is not convincible enough to stress such an important issue. Therefore, we have incorporated additional codon usage indices, SiD, to strength our findings and further evaluate the adaptation of PEDV to host species. We found that the mean SiD value of genotype II was statistically significantly higher compared with genotype I (Figure 9), indicating that host animal (*Sus scrofa*) exerted a significantly deeper selection pressure on Genotype II strains compared to the genotype I counterpart. The RCDI and SiD analyses suggest that PEDV might be adapted to its natural host (*Sus scrofa*), which supporting that *Sus scrofa* plays an important role as a PEDV reservoir (Lee et al., 2016; Turlewicz-Podbielska and Pomorska-Mól, 2021). In addition, those results also reflect that the selection pressure from *Sus scrofa* may influence the codon usage pattern of PEDV and that the translation resources of host system are more efficient for PEDV evolution. The current fact is that PEDV has now spread all around the world, and swine is the only natural host, it is possible that PEDV might improve its adaptive fitness to pig cells through the complex adaptive evolution process, thus resulting in further risks of global transmission and subsequent outbreak. In this respect, stricter anti-PEDV measures are urgently needed.

Up to now, based on the continued study of codon usage patterns of viral genome, we have a considerable understanding of the codon usage profile and the preferred and avoided codons and codon pairs in certain viral coding sequence, and this concept has been successfully applied in synthetic attenuated virus engineering and poliovirus live-attenuated vaccines development (Burns et al., 2006; Coleman et al., 2008). Back to our research, those results we obtained in this study concerning the preferred and under-represented codons in PEDV might be employed for the design of new generation PEDV vaccine and other prevention measures.

Collectively, our study showed that PEDV genome presented a relative low CUB, which suggested that the frequency of synonymous codon usage in PEDV genome is scattered. In addition, two phylogroups of PEDV may evolve with subtle difference under mutation and selection pressures. Moreover, mutation pressure and natural selection are the two main forces in influencing the PEDV’s codon usage pattern, and dinucleotide composition and geographical distribution are another potential influencing factor shaping the codon usage pattern of PEDV. Furthermore, PEDV has evolved a mixture of coincident and antagonistic codon usage patterns relative to *Sus scrofa*, which benefit its host adaptation and viral replicative fitness. This study not only provides a comprehensive investigation of the codon usage patterns of PEDV, but also helps to deepen our understanding of the processes governing the evolution of PEDV.

## Data Availability Statement

The datasets presented in this study can be found in online repositories. The names of the repository/repositories and accession number(s) can be found in the article/Supplementary Material.

## Author Contributions

FS: conceptualization, resources and data curation, writing—original draft preparation, and project administration. FS and LJ: methodology, software, visualization, formal analysis, and investigation. FS, LJ, and WW: validation. FS, WW, and ZL: writing—review and editing. FS and ZL: supervision. FS and RY: funding acquisition. All authors have read and agreed to the published version of the manuscript.

## Conflict of Interest

The authors declare that the research was conducted in the absence of any commercial or financial relationships that could be construed as a potential conflict of interest.

## Publisher’s Note

All claims expressed in this article are solely those of the authors and do not necessarily represent those of their affiliated organizations, or those of the publisher, the editors and the reviewers. Any product that may be evaluated in this article, or claim that may be made by its manufacturer, is not guaranteed or endorsed by the publisher.

## Footnotes

1
http://www.ncbi.nlm.nih.gov/

2
https://www.ebi.ac.uk/Tools/msa/muscle/

3
http://itol.embl.de/

4
https://www.r-project.org/

5
https://www.kazusa.or.jp/codon/

6
https://discover.nci.nih.gov/cimminer/

## Supplementary Material

Supplementary Figure 1 Cluster analysis (Heat map) of RSCU values among different subgenotypes **(A)** or countries **(B)** of PEDV isolates used in this study. The heat map represents the RSCU values divided into 3 ranges: < 1 (Green color), 1–1.6 (Yellow color) and > 1.6 (Distinct red). The heatmap analysis was performed using CIMminer. Each column represents a codon. The higher RSCU value, suggesting more frequent codon usage, was represented with distinct red. The codon usage is highly biased toward A/U-ending codons. Euclidean distance and complete-linkage methods were used to produce the clusters.

Supplementary Table 1 The detailed information describing the 56 PEDV strains used in this study.

Supplementary Table 2 Overall occurrence of nucleotide composition, the third position nucleotide contents and ENC values of 56 PEDV strains.

Supplementary Table 3 Nucleotide composition and properties of the G1 and G2 complete PEDV coding sequences.

Supplementary Table 4 Relative dinucleotide abundance of the complete PEDV coding sequences used in this study.

Supplementary Table 5 AU bias [A3%/(A3% + U3%)] and GC bias [G3%/(G3%+ C3%)] for the 56 PEDV strains used in this study.

## Abbreviations

PEDV: porcine epidemic diarrhea virus
CUB: codon usage bias
RNP: ribonucleoprotein
G1: genotype I
G2: genotype II
ORFs: open reading frames
NJ: neighbor-Joining
ML: maximum-likelihood
GC1s: guanine plus cytosine content of first codon position
GC2s: guanine plus cytosine content of second codon position
GC3s: guanine plus cytosine content of third codon position
ENC: effective codon number
RSCU: relative synonymous codon usage
COA: correspondence analysis
PCA: principal component analysis
PR2: parity rule 2
GRAVY: hydropathicity
ARO: aromaticity
RCDI: relative codon deoptimization index
SiD: similarity index
SD: standard deviation
AA: amino acids
TLR9: toll like receptor 9
SARS-CoV-2: novel severe acute respiratory syndrome coronavirus 2
TGEV: transmissible gastroenteritis virus.
