# Supplementary figures and images for: Study on the Characteristic Codon Usage Pattern in Porcine Epidemic Diarrhea Virus Genomes and Its Host Adaptation Phenotype
Source: Front Microbiol. 2021 Oct 18;12:738082. doi: 10.3389/fmicb.2021.738082 (PMC8558211; doi:10.3389/fmicb.2021.738082)

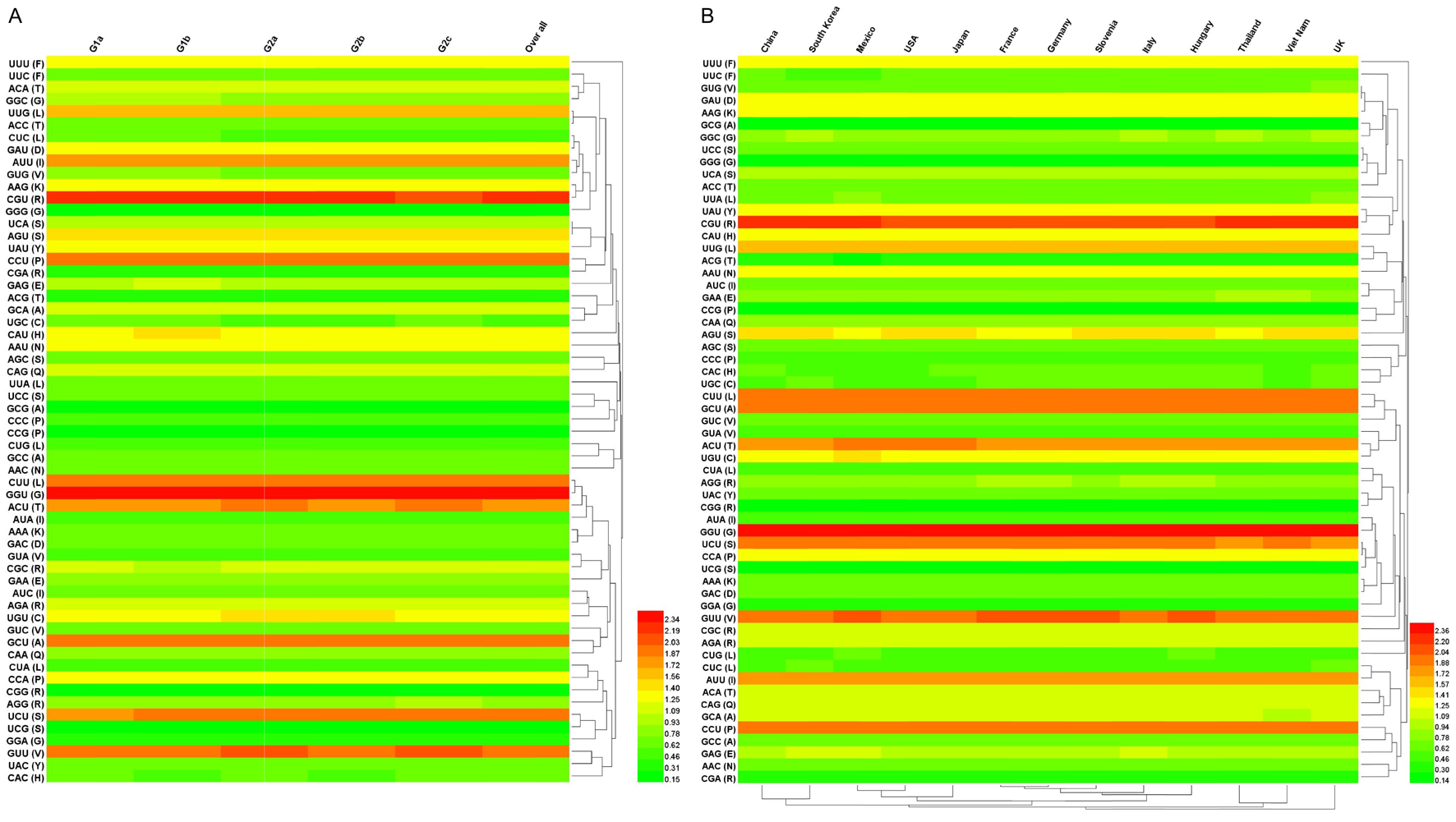

Supplement: Supplementary Figure 1 — Cluster analysis (Heat map) of RSCU values among different subgenotypes (A) or countries (B) of PEDV isolates used in this study. The heat map represents the RSCU values divided into 3 ranges: < 1 (Green color), 1–1.6 (Yellow color) and > 1.6 (Distinct red). The heatmap analysis was performed using CIMminer. Each column represents a codon. The higher RSCU value, suggesting more frequent codon usage, was represented with distinct red. The codon usage is highly biased toward A/U-ending codons. Euclidean distance and complete-linkage methods were used to produce the clusters. [file Image_1.tif]
