# Supplementary material for: Study on the Characteristic Codon Usage Pattern in Porcine Epidemic Diarrhea Virus Genomes and Its Host Adaptation Phenotype
Source: Front Microbiol. 2021 Oct 18;12:738082. doi: 10.3389/fmicb.2021.738082 (PMC8558211; doi:10.3389/fmicb.2021.738082)
Supplement: Supplementary Table 1 — The detailed information describing the 56 PEDV strains used in this study. [file Table_1.DOCX]

**Supplementary Table 1.** The detailed information describing the 56 PEDV strains used in this study.

| **No.** | **Strain** | **Accession Number** | **Date** | **Country** |
| --- | --- | --- | --- | --- |
| 1 | CV777 | AF353511 | 1978 | UK/G1a |
| 2 | CH/S | JN547228 | 1986 | China/G1b |
| 3 | Attenuated CV777 | KT323979 | 1998 | China/G1b |
| 4 | Attenuated DR13 | JQ023162 | N.A. | South Korea/G1a |
| 5 | KUPE21 | MF737355 | Jul-2001 | South Korea/G2b |
| 6 | DR13 | JQ023161 | 2009 | South Korea/G1a |
| 7 | AJ1102 | JX188454 | May-2011 | China/G2b |
| 8 | GD-1 | JX647847 | Dec-2011 | China/G2b |
| 9 | AH2012 | KC210145 | Mar-2012 | China/G2a |
| 10 | SD-M | JX560761 | Feb-2012 | China/G1b |
| 11 | FL2013 | KP765609 | Jul-2013 | China/G2b |
| 12 | YN1 | KT021227 | Aug-2013 | China/G2b |
| 13 | USA/Iowa106/2013 | KJ645695 | Dec-2013 | USA/Indel-like strain/G2c |
| 14 | VN/KCHY-310113/2013 | KJ960180 | 2013 | Viet Nam/G2a |
| 15 | PC21A | KR078299 | Jun-2013 | USA/G2b |
| 16 | PC22A | KY499262 | Jun-2013 | USA/G2a |
| 17 | MEX/104/2013 | KJ645708 | Nov-2013 | Mexico/G2a |
| 18 | USA-Ohio75-2013 | KJ645670 | Nov-2013 | USA/G2a |
| 19 | CBR1 | KR610993 | Jul-2014 | Thailand/G2b |
| 20 | EAS1 | KR610991 | Oct-2014 | Thailand/G1a |
| 21 | HUA-14PED96 | KT941120 | Oct-2014 | Viet Nam/G2a |
| 22 | KCH-2/JPN/2014 | LC063847 | Mar-2014 | Japan/G2a |
| 23 | Tottori2/JPN/2014 | LC022792 | Oct-2014 | Japan/G2a |
| 24 | FR/001/2014 | KR011756 | Dec-2014 | France/Indel-like strain/G2c |
| 25 | OH851 | KJ399978 | Jan-2014 | USA/Indel-like strain/G2c |
| 26 | GER/L00719/2014 | LM645058 | 2014 | Germany/Indel-like strain/G2c |
| 27 | ZL29 | KU847996 | Jul-2015 | China/Indel-like strain/G2c |
| 28 | PEDV/USA/Missouri130/2015 | KU982975 | Oct-2015 | USA/G2a |
| 29 | PEDV/USA/Minnesota125/2015 | KU982980 | Oct-2015 | USA/G2a |
| 30 | PEDV/USA/NorthDakota93/2015 | KU982970 | Nov-2015 | USA/G2a |
| 31 | SLO/JH-11/2015 | KU297956 | Sep-2015 | Slovenia/G2b |
| 32 | SLOreBAS-1/2015 | KY019623 | Nov-2015 | Slovenia/G2c |
| 33 | JSLS-1/2015 | KX534205 | Jan-2015 | China/G1b |
| 34 | JS-2/2015 | KX534206 | Jan-2015 | China/G1b |
| 35 | CH/HNAY/2015 | KR809885 | Mar-2015 | China/G2a |
| 36 | CH/HNQX-3/14 | KR095279 | Jan-2015 | China/G2a |
| 37 | PEDV/USA/Iowa127/2015 | KU982969 | Oct-2015 | USA/G2a |
| 38 | PEDV/USA/Minnesota124/2015 | KU982981 | Oct-2015 | USA/G2a |
| 39 | HM2017 | MK690502 | Mar-2016 | China/G2a |
| 40 | PEDV SH | MK841494 | Oct-2016 | China/G2b |
| 41 | PEDV1842/2016 ITA | KY111278 | Jan-2016 | Italy/G2c |
| 42 | KNU-1706 | MH052685 | Dec-2017 | South Korea/G2a |
| 43 | PEDV JS-A | MH748550 | Nov-2017 | China/G2b |
| 44 | PEDV/MEX/QRO/02/2017 | MH013466 | Jun-2017 | Mexico/G2b |
| 45 | TC-PC177 | KY499261 | 2017 | USA (197 aa deletion)/G2a |
| 46 | USA/OK10240-8/2017 | MG334555 | Feb-2017 | USA (200 aa deletion) /G2a |
| 47 | C3-HB2017 | MF807951 | Feb-2017 | China/G2a |
| 48 | CH/SCGA/2017 | MH061336 | Jan-2017 | China/G2a |
| 49 | CH/SCLS/2018 | MH061341 | Jan-2018 | China/G2c |
| 50 | CH/SCZJ/2018 | MH061342 | Feb-2018 | China/G2a |
| 51 | CH/SCMY/2018 | MH061343 | Jan-2018 | China/G2a |
| 52 | TW/Yunlin550/2018 | MK673545 | Feb-2018 | China, Taiwan/G2b |
| 53 | V7-HB2018 | MK138516 | Mar-2018 | China/G2a |
| 54 | CN/Liaoning25/2018 | MK796238 | Mar-2018 | China/G1b |
| 55 | S236 | MH593900 | Mar-2018 | Hungary/G2c |
| 56 | CT P10 | MN114121 | Mar-2018 | China/G2c |

**Note**: N.A., not available.
