# Supplementary material for: Study on the Characteristic Codon Usage Pattern in Porcine Epidemic Diarrhea Virus Genomes and Its Host Adaptation Phenotype
Source: Front Microbiol. 2021 Oct 18;12:738082. doi: 10.3389/fmicb.2021.738082 (PMC8558211; doi:10.3389/fmicb.2021.738082)
Supplement: Supplementary Table 2 — Overall occurrence of nucleotide composition, the third position nucleotide contents and ENC values of 56 PEDV strains. [file Table_2.DOCX]

**Supplementary Table 2.** Overall occurrence of nucleotide composition, the third position nucleotide contents and ENC values of 56 PEDV strains.

| **No.*^a^*** | **A** | **T** | **G** | **C** | **A3** | **T3** | **G3** | **C3** | **GC3** | **ENC^a^** | **A+U** | **G+C** |
| --- | --- | --- | --- | --- | --- | --- | --- | --- | --- | --- | --- | --- |
| 1 | 0.2481 | 0.3318 | 0.2289 | 0.1912 | 0.2369 | 0.537 | 0.2297 | 0.233 | 0.359 | 48.36 | 0.58 | 0.42 |
| 2 | 0.2487 | 0.3339 | 0.2274 | 0.1901 | 0.2372 | 0.5431 | 0.2279 | 0.2296 | 0.354 | 48.14 | 0.583 | 0.417 |
| 3 | 0.2476 | 0.3324 | 0.2294 | 0.1906 | 0.2356 | 0.5394 | 0.2311 | 0.2314 | 0.358 | 48.19 | 0.58 | 0.42 |
| 4 | 0.2482 | 0.3330 | 0.229 | 0.1897 | 0.2365 | 0.5409 | 0.2307 | 0.2303 | 0.357 | 48.05 | 0.581 | 0.419 |
| 5 | 0.2488 | 0.3330 | 0.2277 | 0.1906 | 0.2388 | 0.5414 | 0.2257 | 0.2308 | 0.354 | 48.29 | 0.582 | 0.418 |
| 6 | 0.2479 | 0.3334 | 0.2286 | 0.1901 | 0.2355 | 0.5423 | 0.2304 | 0.2289 | 0.356 | 48.05 | 0.581 | 0.419 |
| 7 | 0.2486 | 0.3339 | 0.2275 | 0.1900 | 0.238 | 0.5445 | 0.2264 | 0.2284 | 0.352 | 47.96 | 0.582 | 0.418 |
| 8 | 0.2485 | 0.3340 | 0.2276 | 0.1900 | 0.2386 | 0.5438 | 0.2259 | 0.2288 | 0.352 | 47.91 | 0.582 | 0.418 |
| 9 | 0.2281 | 0.3331 | 0.2271 | 0.1917 | 0.2386 | 0.5428 | 0.2235 | 0.2314 | 0.353 | 48.14 | 0.581 | 0.419 |
| 10 | 0.2481 | 0.3333 | 0.2289 | 0.1897 | 0.2363 | 0.5408 | 0.2309 | 0.2302 | 0.357 | 48.06 | 0.581 | 0.419 |
| 11 | 0.2484 | 0.3341 | 0.2277 | 0.1898 | 0.2376 | 0.5445 | 0.2267 | 0.2285 | 0.352 | 47.92 | 0.583 | 0.417 |
| 12 | 0.2284 | 0.3342 | 0.2276 | 0.1897 | 0.2381 | 0.5449 | 0.2262 | 0.2281 | 0.352 | 47.96 | 0.583 | 0.417 |
| 13 | 0.2490 | 0.3334 | 0.2271 | 0.1904 | 0.2393 | 0.5421 | 0.2253 | 0.2308 | 0.353 | 48.04 | 0.582 | 0.418 |
| 14 | 0.2483 | 0.3331 | 0.2283 | 0.1903 | 0.2377 | 0.542 | 0.227 | 0.2297 | 0.354 | 48.22 | 0.581 | 0.419 |
| 15 | 0.2490 | 0.3337 | 0.2270 | 0.1903 | 0.2394 | 0.5444 | 0.2241 | 0.229 | 0.351 | 47.95 | 0.583 | 0.417 |
| 16 | 0.2489 | 0.3337 | 0.2270 | 0.1903 | 0.2394 | 0.5443 | 0.2243 | 0.229 | 0.351 | 47.96 | 0.583 | 0.417 |
| 17 | 0.2487 | 0.3334 | 0.2270 | 0.1901 | 0.239 | 0.5453 | 0.2242 | 0.2284 | 0.351 | 47.94 | 0.583 | 0.417 |
| 18 | 0.2487 | 0.3338 | 0.2271 | 0.1904 | 0.2392 | 0.544 | 0.2244 | 0.2293 | 0.352 | 48.05 | 0.583 | 0.417 |
| 19 | 0.2486 | 0.3328 | 0.2279 | 0.1907 | 0.2382 | 0.5415 | 0.226 | 0.2308 | 0.354 | 48.23 | 0.581 | 0.419 |
| 20 | 0.2485 | 0.3311 | 0.2290 | 0.1914 | 0.2383 | 0.5351 | 0.2312 | 0.2337 | 0.36 | 48.63 | 0.58 | 0.42 |
| 21 | 0.2487 | 0.3343 | 0.2269 | 0.1902 | 0.2397 | 0.5437 | 0.2244 | 0.2288 | 0.351 | 48.07 | 0.583 | 0.417 |
| 22 | 0.2488 | 0.3335 | 0.2272 | 0.1906 | 0.2384 | 0.5428 | 0.2258 | 0.23 | 0.353 | 48.04 | 0.582 | 0.418 |
| 23 | 0.2487 | 0.3343 | 0.2273 | 0.1898 | 0.2399 | 0.5436 | 0.2258 | 0.2281 | 0.352 | 48.03 | 0.583 | 0.417 |
| 24 | 0.2488 | 0.3334 | 0.2273 | 0.1904 | 0.2393 | 0.5416 | 0.2257 | 0.2307 | 0.354 | 48.21 | 0.582 | 0.418 |
| 25 | 0.2487 | 0.3338 | 0.2271 | 0.1903 | 0.2386 | 0.5437 | 0.2252 | 0.2298 | 0.353 | 48.04 | 0.583 | 0.417 |
| 26 | 0.2488 | 0.3334 | 0.2273 | 0.1905 | 0.239 | 0.5419 | 0.2258 | 0.2305 | 0.354 | 48.15 | 0.582 | 0.418 |
| 27 | 0.2485 | 0.3328 | 0.2276 | 0.1911 | 0.2396 | 0.5401 | 0.225 | 0.232 | 0.355 | 48.34 | 0.581 | 0.419 |
| 28 | 0.2484 | 0.3346 | 0.2268 | 0.1902 | 0.2381 | 0.5462 | 0.2242 | 0.2279 | 0.35 | 47.9 | 0.583 | 0.417 |
| 29 | 0.2486 | 0.3347 | 0.2267 | 0.1898 | 0.2396 | 0.5453 | 0.2236 | 0.2279 | 0.35 | 47.88 | 0.583 | 0.417 |
| 30 | 0.2487 | 0.3342 | 0.2270 | 0.1900 | 0.2393 | 0.5453 | 0.2243 | 0.228 | 0.35 | 47.97 | 0.583 | 0.417 |
| 31 | 0.2488 | 0.3336 | 0.2272 | 0.1903 | 0.2394 | 0.5417 | 0.2254 | 0.2304 | 0.353 | 48.21 | 0.582 | 0.418 |
| 32 | 0.2485 | 0.3335 | 0.2273 | 0.1907 | 0.239 | 0.5421 | 0.2254 | 0.2302 | 0.353 | 48.24 | 0.582 | 0.418 |
| 33 | 0.2479 | 0.3333 | 0.2292 | 0.1897 | 0.2362 | 0.5406 | 0.2307 | 0.2305 | 0.357 | 48.04 | 0.581 | 0.419 |
| 34 | 0.2481 | 0.3332 | 0.2290 | 0.1897 | 0.2362 | 0.5409 | 0.231 | 0.2303 | 0.357 | 48.06 | 0.581 | 0.419 |
| 35 | 0.2488 | 0.3326 | 0.2274 | 0.1912 | 0.2401 | 0.5402 | 0.2252 | 0.2313 | 0.354 | 48.39 | 0.581 | 0.419 |
| 36 | 0.2479 | 0.3325 | 0.2279 | 0.1918 | 0.2378 | 0.5386 | 0.2272 | 0.2331 | 0.357 | 48.46 | 0.58 | 0.42 |
| 37 | 0.2489 | 0.3342 | 0.2269 | 0.1900 | 0.2395 | 0.5451 | 0.2242 | 0.2281 | 0.35 | 47.99 | 0.583 | 0.417 |
| 38 | 0.2487 | 0.3340 | 0.2271 | 0.1902 | 0.2392 | 0.5451 | 0.2242 | 0.2281 | 0.351 | 48 | 0.583 | 0.417 |
| 39 | 0.2488 | 0.3335 | 0.2274 | 0.1903 | 0.2392 | 0.5434 | 0.2252 | 0.2291 | 0.352 | 48.18 | 0.582 | 0.418 |
| 40 | 0.2484 | 0.3340 | 0.2273 | 0.1903 | 0.2385 | 0.5449 | 0.2249 | 0.2283 | 0.351 | 48 | 0.582 | 0.418 |
| 41 | 0.2488 | 0.3331 | 0.2275 | 0.1907 | 0.2387 | 0.5407 | 0.2261 | 0.2314 | 0.355 | 48.3 | 0.582 | 0.418 |
| 42 | 0.249 | 0.334 | 0.227 | 0.1899 | 0.2397 | 0.5455 | 0.2244 | 0.2277 | 0.35 | 47.93 | 0.583 | 0.417 |
| 43 | 0.2486 | 0.3341 | 0.2274 | 0.1897 | 0.2392 | 0.5439 | 0.226 | 0.2284 | 0.352 | 47.89 | 0.583 | 0.417 |
| 44 | 0.2488 | 0.3347 | 0.2269 | 0.1897 | 0.2397 | 0.5456 | 0.2237 | 0.2281 | 0.35 | 47.93 | 0.583 | 0.417 |
| 45 | 0.2487 | 0.3342 | 0.2273 | 0.1899 | 0.2395 | 0.5435 | 0.2259 | 0.2285 | 0.352 | 47.94 | 0.583 | 0.417 |
| 46 | 0.2483 | 0.3344 | 0.2272 | 0.1900 | 0.2386 | 0.5444 | 0.2257 | 0.228 | 0.352 | 47.91 | 0.583 | 0.417 |
| 47 | 0.2486 | 0.3325 | 0.2276 | 0.1913 | 0.24 | 0.5411 | 0.2244 | 0.23 | 0.353 | 48.33 | 0.581 | 0.419 |
| 48 | 0.2489 | 0.3333 | 0.2270 | 0.1909 | 0.2394 | 0.5421 | 0.2242 | 0.2309 | 0.353 | 48.11 | 0.582 | 0.418 |
| 49 | 0.2490 | 0.3332 | 0.2268 | 0.1909 | 0.2395 | 0.5436 | 0.2234 | 0.2303 | 0.352 | 48 | 0.582 | 0.418 |
| 50 | 0.2485 | 0.3344 | 0.2271 | 0.1900 | 0.2382 | 0.5445 | 0.2254 | 0.2289 | 0.352 | 47.85 | 0.583 | 0.417 |
| 51 | 0.2488 | 0.3337 | 0.2270 | 0.1905 | 0.2394 | 0.5424 | 0.2247 | 0.2305 | 0.353 | 48.15 | 0.582 | 0.418 |
| 52 | 0.2488 | 0.3340 | 0.2272 | 0.1901 | 0.2388 | 0.543 | 0.2261 | 0.2296 | 0.353 | 48.06 | 0.583 | 0.417 |
| 53 | 0.2490 | 0.3336 | 0.2269 | 0.1905 | 0.2403 | 0.5433 | 0.2231 | 0.23 | 0.352 | 48.12 | 0.583 | 0.417 |
| 54 | 0.2480 | 0.3336 | 0.2286 | 0.1898 | 0.2369 | 0.5415 | 0.2305 | 0.2291 | 0.356 | 48.05 | 0.582 | 0.418 |
| 55 | 0.2485 | 0.3330 | 0.2276 | 0.1909 | 0.2389 | 0.5404 | 0.2261 | 0.2313 | 0.355 | 48.38 | 0.581 | 0.419 |
| 56 | 0.2485 | 0.3333 | 0.2273 | 0.1909 | 0.2389 | 0.5417 | 0.2243 | 0.2317 | 0.354 | 48.18 | 0.58 | 0.42 |
| **Mean** | 0.2478  ±0.004 | 0.3335  ±0.00 | 0.2275  ±0.00 | 0.1904  ±0.00 | 0.2386  ±0.001 | 0.5426  ±0.002 | 0.2261  ±0.002 | 0.2298  ±0.001 | 0.3533  ±0.002 | 48.1  ±0.166 | 0.582  ±0.000 | 0.4179  ±0.000 |

**Note**: A, U, G, and C represent the composition of A, U, G, and C in the complete PEDV coding sequences, respectively; A3s, U3s, C3s, and G3s represent the content of A, U, C, and G at the third codon positions; ENC, the effective codon number.

*^a^* Numbers (1-56) are corresponding to the sequence order in Table S1.
