# Supplementary material for: Study on the Characteristic Codon Usage Pattern in Porcine Epidemic Diarrhea Virus Genomes and Its Host Adaptation Phenotype
Source: Front Microbiol. 2021 Oct 18;12:738082. doi: 10.3389/fmicb.2021.738082 (PMC8558211; doi:10.3389/fmicb.2021.738082)
Supplement: Supplementary Table 3 — Nucleotide composition and properties of the G1 and G2 complete PEDV coding sequences. [file Table_3.DOCX]

**Supplementary Table 3.** Nucleotide composition and properties of the G1 and G2 complete PEDV coding sequences.

| **Categories** | **G1** | | **G2** | | | **All** |
| --- | --- | --- | --- | --- | --- | --- |
|  | **1a** | **1b** | **2a** | **2b** | **2c** |  |
| A | 0.2482±0.000 | 0.2481±0.000 | 0.2478±0.004 | 0.247±0.006 | 0.2487±0.000 | 0.2478±0.004 |
| U | 0.3323±0.001 | 0.3333±0.000 | 0.3337±0.001 | 0.3338±0.001 | 0.3333±0.006 | 0.3335±0.001 |
| G | 0.2289±0.000 | 0.2288±0.000 | 0.2272±0.000 | 0.2274±0.000 | 0.2273±0.006 | 0.2275±0.001 |
| C | 0.1906±0.000 | 0.1899±0.000 | 0.1904±0.001 | 0.1901±0.000 | 0.1907±0.006 | 0.1904±0.001 |
| A3 | 0.2368±0.001 | 0.2364±0.000 | 0.2392±0.001 | 0.2387±0.001 | 0.2391±0.006 | 0.2386±0.001 |
| U3 | 0.5388±0.003 | 0.5411±0.001 | 0.5435±0.002 | 0.5437±0.001 | 0.5418±0.001 | 0.5426±0.002 |
| G3 | 0.2305±0.000 | 0.2304±0.001 | 0.2248±0.001 | 0.2256±0.001 | 0.2252±0.001 | 0.2261±0.002 |
| C3 | 0.2315±0.002 | 0.2302±0.000 | 0.2293±0.001 | 0.2291±0.001 | 0.2309±0.001 | 0.2298±0.001 |
| AU | 0.5805±0.000 | 0.5813±0.001 | 0.5824±0.001 | 0.5824±0.001 | 0.5817±0.001 | 0.5820±0.001 |
| GC | 0.4195±0.000 | 0.4187±0.001 | 0.4176±0.001 | 0.4176±0.001 | 0.4183±0.001 | 0.4179±0.001 |
| GC1s | 0.491±0.000 | 0.4909±0.001 | 0.4908±0.001 | 0.4904±0.000 | 0.4903±0.001 | 0.4907±0.001 |
| GC2s | 0.3899±0.000 | 0.3888±0.000 | 0.3901±0.001 | 0.39±0.001 | 0.3902±0.001 | 0.3899±0.001 |
| GC3s | 0.3777±0.002 | 0.3763±0.001 | 0.3719±0.002 | 0.3721±0.001 | 0.3735±0.001 | 0.3731±0.002 |
| GC12s | 0.4404±0.000 | 0.4390±0.002 | 0.4405±0.000 | 0.4902±0.000 | 0.4902±0.000 | 0.4902±0.000 |
| ENC | 48.27±0.270 | 48.09±0.061 | 48.07±0.162 | 48.03±0.139 | 48.19±0.132 | 48.1±0.166 |

**Note:** A, U, G, and C represent the average composition of A, U, G, and C in the complete PEDV coding sequences of corresponding genotypes; GC1s, GC2s, and GC3s represent the GC content at the first, second and third codon positions, respectively, and GC12s represents the mean value of GC1s and GC2s. A3, U3, C3, and G3 represent the content of A, U, C, and G at the third codon positions. The mean value of each category was listed on the right column.
