# Supplementary material for: Study on the Characteristic Codon Usage Pattern in Porcine Epidemic Diarrhea Virus Genomes and Its Host Adaptation Phenotype
Source: Front Microbiol. 2021 Oct 18;12:738082. doi: 10.3389/fmicb.2021.738082 (PMC8558211; doi:10.3389/fmicb.2021.738082)
Supplement: Supplementary Table 4 — Relative dinucleotide abundance of the complete PEDV coding sequences used in this study. [file Table_4.DOCX]

**Supplementary Table 4.** Relative dinucleotide abundance of the complete PEDV coding sequences used in this study.

| **Categories** | **G1** | | **G2** | | | **All** |
| --- | --- | --- | --- | --- | --- | --- |
|  | **1a** | **1b** | **2a** | **2b** | **2c** |  |
| **UU** | 1.006±0.004 | 1.004±0.001 | 1.006±0.002 | 1.006±0.003 | 1.007±0.002 | 1.006±0.002 |
| **UG** | 1.330±0.005 | 1.336±0.001 | 1.330±0.003 | 1.329±0.003 | 1.331±0.002 | 1.331±0.003 |
| **GU** | 1.072±0.004 | 1.071±0.002 | 1.081±0.003 | 1.079±0.004 | 1.078±0.002 | 1.078±0.004 |
| **AU** | 0.863±0.005 | 0.869±0.002 | 0.854±0.002 | 0.856±0.002 | 0.851±0.002 | 0.856±0.005 |
| **UA** | 0.852±0.005 | 0.849±0.001 | 0.850±0.002 | 0.853±0.003 | 0.849±0.002 | 0.851±0.003 |
| **CU** | 1.081±0.003 | 1.080±0.001 | 1.089±0.003 | 1.086±0.003 | 1.091±0.003 | 1.087±0.004 |
| **AA** | 1.059±0.006 | 1.056±0.002 | 1.060±0.003 | 1.060±0.004 | 1.060±0.002 | 1.059±0.004 |
| **CA** | 1.353±0.006 | 1.359±0.004 | 1.349±0.004 | 1.348±0.005 | 1.355±0.005 | 1.351±0.006 |
| **AC** | 1.201±0.007 | 1.197±0.004 | 1.214±0.003 | 1.212±0.003 | 1.216±0.003 | 1.211±0.007 |
| **AG** | 0.968±0.003 | 0.966±0.002 | 0.975±0.004 | 0.972±0.005 | 0.974±0.001 | 0.973±0.004 |
| **UC** | 0.787±0.003 | 0.786±0.003 | 0.786±0.003 | 0.784±0.003 | 0.786±0.002 | 0.786±0.003 |
| **GC** | 1.115±0.008 | 1.121±0.006 | 1.103±0.004 | 1.110±0.005 | 1.104±0.003 | 1.107±0.008 |
| **GA** | 0.861±0.006 | 0.861±0.001 | 0.857±0.004 | 0.856±0.004 | 0.855±0.002 | 0.857±0.004 |
| **GG** | 0.923±0.003 | 0.931±0.002 | 0.924±0.003 | 0.928±0.003 | 0.926±0.002 | 0.926±0.004 |
| **CC** | 0.945±0.007 | 0.949±0.003 | 0.933±0.007 | 0.940±0.007 | 0.924±0.003 | 0.936±0.100 |
| **CG** | 0.547±0.008 | 0.537±0.004 | 0.544±0.005 | 0.543±0.003 | 0.543±0.004 | 0.543±0.005 |
