# Supplementary material for: Study on the Characteristic Codon Usage Pattern in Porcine Epidemic Diarrhea Virus Genomes and Its Host Adaptation Phenotype
Source: Front Microbiol. 2021 Oct 18;12:738082. doi: 10.3389/fmicb.2021.738082 (PMC8558211; doi:10.3389/fmicb.2021.738082)
Supplement: Supplementary Table 5 — AU bias [A3%/(A3% + U3%)] and GC bias [G3%/(G3%+ C3%)] for the 56 PEDV strains used in this study. [file Table_5.DOCX]

**Supplementary Table 5.** AU bias [A3%/(A3% + U3%)] and GC bias [G3%/(G3%+ C3%)] for the 56 PEDV strains used in this study.

| **No.** | **Strain** | **Accession Number** | **U3s** | **C3s** | **A3s** | **G3s** | **A3+U3** | **G3+C3** | **A3/(A3+U3)** | **G3/(G3+C3)** |
| --- | --- | --- | --- | --- | --- | --- | --- | --- | --- | --- |
| 1 | CV777 | AF353511 | 0.537 | 0.233 | 0.2369 | 0.2297 | 0.7739 | 0.4627 | 0.3061 | 0.4964 |
| 2 | CH/S | JN547228 | 0.5431 | 0.2296 | 0.2372 | 0.2279 | 0.7803 | 0.4575 | 0.3040 | 0.4981 |
| 3 | Attenuated CV777 | KT323979 | 0.5394 | 0.2314 | 0.2356 | 0.2311 | 0.775 | 0.4625 | 0.3040 | 0.4997 |
| 4 | Attenuated DR13 | JQ023162 | 0.5409 | 0.2303 | 0.2365 | 0.2307 | 0.7774 | 0.461 | 0.3042 | 0.5004 |
| 5 | KUPE21 | MF737355 | 0.5414 | 0.2308 | 0.2388 | 0.2257 | 0.7802 | 0.4565 | 0.3061 | 0.4944 |
| 6 | DR13 | JQ023161 | 0.5423 | 0.2289 | 0.2355 | 0.2304 | 0.7778 | 0.4593 | 0.3028 | 0.5016 |
| 7 | AJ1102 | JX188454 | 0.5445 | 0.2284 | 0.238 | 0.2264 | 0.7825 | 0.4548 | 0.3042 | 0.4978 |
| 8 | GD-1 | JX647847 | 0.5438 | 0.2288 | 0.2386 | 0.2259 | 0.7824 | 0.4547 | 0.3050 | 0.4968 |
| 9 | AH2012 | KC210145 | 0.5428 | 0.2314 | 0.2386 | 0.2235 | 0.7814 | 0.4549 | 0.3053 | 0.4913 |
| 10 | SD-M | JX560761 | 0.5408 | 0.2302 | 0.2363 | 0.2309 | 0.7771 | 0.4611 | 0.3041 | 0.5008 |
| 11 | FL2013 | KP765609 | 0.5445 | 0.2285 | 0.2376 | 0.2267 | 0.7821 | 0.4552 | 0.3038 | 0.4980 |
| 12 | YN1 | KT021227 | 0.5449 | 0.2281 | 0.2381 | 0.2262 | 0.783 | 0.4543 | 0.3041 | 0.4979 |
| 13 | USA/Iowa106/2013 | KJ645695 | 0.5421 | 0.2308 | 0.2393 | 0.2253 | 0.7814 | 0.4561 | 0.3062 | 0.4940 |
| 14 | VN/KCHY-310113/2013 | KJ960180 | 0.542 | 0.2297 | 0.2377 | 0.227 | 0.7797 | 0.4567 | 0.3049 | 0.4970 |
| 15 | PC21A | KR078299 | 0.5444 | 0.229 | 0.2394 | 0.2241 | 0.7838 | 0.4531 | 0.3054 | 0.4946 |
| 16 | PC22A | KY499262 | 0.5443 | 0.229 | 0.2394 | 0.2243 | 0.7837 | 0.4533 | 0.3055 | 0.4948 |
| 17 | MEX/104/2013 | KJ645708 | 0.5453 | 0.2284 | 0.239 | 0.2242 | 0.7843 | 0.4526 | 0.3047 | 0.4954 |
| 18 | USA-Ohio75-2013 | KJ645670 | 0.544 | 0.2293 | 0.2392 | 0.2244 | 0.7832 | 0.4537 | 0.3054 | 0.4946 |
| 19 | CBR1 | KR610993 | 0.5415 | 0.2308 | 0.2382 | 0.226 | 0.7797 | 0.4568 | 0.3055 | 0.4947 |
| 20 | EAS1 | KR610991 | 0.5351 | 0.2337 | 0.2383 | 0.2312 | 0.7734 | 0.4649 | 0.3081 | 0.4973 |
| 21 | HUA-14PED96 | KT941120 | 0.5437 | 0.2288 | 0.2397 | 0.2244 | 0.7834 | 0.4532 | 0.3060 | 0.4951 |
| 22 | KCH-2/JPN/2014 | LC063847 | 0.5428 | 0.23 | 0.2384 | 0.2258 | 0.7812 | 0.4558 | 0.3052 | 0.4954 |
| 23 | Tottori2/JPN/2014 | LC022792 | 0.5436 | 0.2281 | 0.2399 | 0.2258 | 0.7835 | 0.4539 | 0.3062 | 0.4975 |
| 24 | FR/001/2014 | KR011756 | 0.5416 | 0.2307 | 0.2393 | 0.2257 | 0.7809 | 0.4564 | 0.3064 | 0.4945 |
| 25 | OH851 | KJ399978 | 0.5437 | 0.2298 | 0.2386 | 0.2252 | 0.7823 | 0.455 | 0.3050 | 0.4949 |
| 26 | GER/L00719/2014 | LM645058 | 0.5419 | 0.2305 | 0.239 | 0.2258 | 0.7809 | 0.4563 | 0.3061 | 0.4948 |
| 27 | ZL29 | KU847996 | 0.5401 | 0.232 | 0.2396 | 0.225 | 0.7797 | 0.457 | 0.3073 | 0.4923 |
| 28 | PEDV/USA/Missouri130/2015 | KU982975 | 0.5462 | 0.2279 | 0.2381 | 0.2242 | 0.7843 | 0.4521 | 0.3036 | 0.4959 |
| 29 | PEDV/USA/Minnesota125/2015 | KU982980 | 0.5453 | 0.2279 | 0.2396 | 0.2236 | 0.7849 | 0.4515 | 0.3053 | 0.4952 |
| 30 | PEDV/USA/NorthDakota93/2015 | KU982970 | 0.5453 | 0.228 | 0.2393 | 0.2243 | 0.7846 | 0.4523 | 0.3050 | 0.4959 |
| 31 | SLO/JH-11/2015 | KU297956 | 0.5417 | 0.2304 | 0.2394 | 0.2254 | 0.7811 | 0.4558 | 0.3065 | 0.4945 |
| 32 | SLOreBAS-1/2015 | KY019623 | 0.5421 | 0.2302 | 0.239 | 0.2254 | 0.7811 | 0.4556 | 0.3060 | 0.4947 |
| 33 | JSLS-1/2015 | KX534205 | 0.5406 | 0.2305 | 0.2362 | 0.2307 | 0.7768 | 0.4612 | 0.3041 | 0.5002 |
| 34 | JS-2/2015 | KX534206 | 0.5409 | 0.2303 | 0.2362 | 0.231 | 0.7771 | 0.4613 | 0.3040 | 0.5008 |
| 35 | CH/HNAY/2015 | KR809885 | 0.5402 | 0.2313 | 0.2401 | 0.2252 | 0.7803 | 0.4565 | 0.3077 | 0.4933 |
| 36 | CH/HNQX-3/14 | KR095279 | 0.5386 | 0.2331 | 0.2378 | 0.2272 | 0.7764 | 0.4603 | 0.3063 | 0.4936 |
| 37 | PEDV/USA/Iowa127/2015 | KU982969 | 0.5451 | 0.2281 | 0.2395 | 0.2242 | 0.7846 | 0.4523 | 0.3053 | 0.4957 |
| 38 | PEDV/USA/Minnesota124/2015 | KU982981 | 0.5451 | 0.2281 | 0.2392 | 0.2242 | 0.7843 | 0.4523 | 0.3050 | 0.4957 |
| 39 | HM2017 | MK690502 | 0.5434 | 0.2291 | 0.2392 | 0.2252 | 0.7826 | 0.4543 | 0.3056 | 0.4957 |
| 40 | PEDV SH | MK841494 | 0.5449 | 0.2283 | 0.2385 | 0.2249 | 0.7834 | 0.4532 | 0.3044 | 0.4962 |
| 41 | PEDV1842/2016 ITA | KY111278 | 0.5407 | 0.2314 | 0.2387 | 0.2261 | 0.7794 | 0.4575 | 0.3063 | 0.4942 |
| 42 | KNU-1706 | MH052685 | 0.5455 | 0.2277 | 0.2397 | 0.2244 | 0.7852 | 0.4521 | 0.3053 | 0.4964 |
| 43 | PEDV JS-A | MH748550 | 0.5439 | 0.2284 | 0.2392 | 0.226 | 0.7831 | 0.4544 | 0.3055 | 0.4974 |
| 44 | PEDV/MEX/QRO/02/2017 | MH013466 | 0.5456 | 0.2281 | 0.2397 | 0.2237 | 0.7853 | 0.4518 | 0.3052 | 0.4951 |
| 45 | TC-PC177 | KY499261 | 0.5435 | 0.2285 | 0.2395 | 0.2259 | 0.783 | 0.4544 | 0.3059 | 0.4971 |
| 46 | USA/OK10240-8/2017 | MG334555 | 0.5444 | 0.228 | 0.2386 | 0.2257 | 0.783 | 0.4537 | 0.3047 | 0.4975 |
| 47 | C3-HB2017 | MF807951 | 0.5411 | 0.23 | 0.24 | 0.2244 | 0.7811 | 0.4544 | 0.3073 | 0.4938 |
| 48 | CH/SCGA/2017 | MH061336 | 0.5421 | 0.2309 | 0.2394 | 0.2242 | 0.7815 | 0.4551 | 0.3063 | 0.4926 |
| 49 | CH/SCLS/2018 | MH061341 | 0.5436 | 0.2303 | 0.2395 | 0.2234 | 0.7831 | 0.4537 | 0.3058 | 0.4924 |
| 50 | CH/SCZJ/2018 | MH061342 | 0.5445 | 0.2289 | 0.2382 | 0.2254 | 0.7827 | 0.4543 | 0.3043 | 0.4961 |
| 51 | CH/SCMY/2018 | MH061343 | 0.5424 | 0.2305 | 0.2394 | 0.2247 | 0.7818 | 0.4552 | 0.3062 | 0.4936 |
| 52 | TW/Yunlin550/2018 | MK673545 | 0.543 | 0.2296 | 0.2388 | 0.2261 | 0.7818 | 0.4557 | 0.3054 | 0.4962 |
| 53 | V7-HB2018 | MK138516 | 0.5433 | 0.23 | 0.2403 | 0.2231 | 0.7836 | 0.4531 | 0.3067 | 0.4924 |
| 54 | CN/Liaoning25/2018 | MK796238 | 0.5415 | 0.2291 | 0.2369 | 0.2305 | 0.7784 | 0.4596 | 0.3043 | 0.5015 |
| 55 | S236 | MH593900 | 0.5404 | 0.2313 | 0.2389 | 0.2261 | 0.7793 | 0.4574 | 0.3066 | 0.4943 |
| 56 | CT P10 | MN114121 | 0.5417 | 0.2317 | 0.2389 | 0.2243 | 0.7806 | 0.456 | 0.3060 | 0.4919 |
| Mean | N. A. | N. A. | 0.5426  ±0.002 | 0.2298  ±0.001 | 0.2386  ±0.001 | 0.2261  ±0.002 | 0.7812  ±0.003 | 0.4558  ±0.003 | 0.3054  ±0.001 | 0.4959  ±0.002 |

**Note**: A3s, U3s, C3s, and G3s represent the content of A, U, C, and G at the third codon positions. N.A., not available.
